# Natural Fiber@MXene‐Engineered Chitosan Aerogels: Thermodynamic‐Transport Synergy for Solar‐Driven Hypersaline Interfacial Evaporation

**DOI:** 10.1002/advs.202505944

**Published:** 2025-05-20

**Authors:** Qin Su, Haidi Wu, Suyang Hou, Liping Ye, Yifan Feng, Longjuan Lu, Biwang Pan, Wancheng Gu, Longcheng Tang, Xuewu Huang, Huaiguo Xue, Jiefeng Gao

**Affiliations:** ^1^ School of Chemistry and Chemical Engineering Yangzhou University No 180, Road Siwangting Yangzhou Jiangsu 225002 China; ^2^ Key Laboratory of Organosilicon Chemistry and Material Technology of Ministry of Education Hangzhou Normal University Building 22, Qinyuan, No.2318, Yuhangtang Road, Cangqian Street, Yuhang District Hangzhou 311121 P. R. China; ^3^ Testing Center Yangzhou University Yangzhou Jiangsu Province 225002 China

**Keywords:** Chitosan aerogel, Kapok fibers, MXene, solar‐driven interfacial evaporation

## Abstract

Enhancing interfacial evaporation rates and optimizing energy utilization remain critical challenges in solar‐driven steam generation. Natural fiber@MXene‐engineered chitosan aerogels with hierarchically oriented channels to achieve high‐efficiency solar‐driven steam generation are developed. The kapok fiber@MXene core–shell units (MKFs) construct photon‐entrapping topological networks that enhance light absorption while simultaneously reinforcing the aerogel's structural integrity and durability for practical applications. The aerogel's oriented microchannels establish thermodynamic potential gradients, facilitating spontaneous capillary‐driven water replenishment and environmental thermal harvesting. Both experimental results and COMSOL multiphysics simulations systematically demonstrate that hierarchical pore channels enhance water transport, improve solar‐thermal/environmental energy synergy, and promote the downward diffusion of concentrated ions from the evaporation surface, achieving an evaporation rate up to 4.40 kg m^−2^ h^−1^ with efficient salt rejection. Long‐term outdoor tests with various corrosive wastewater solutions further validate the aerogel's durability in solar‐driven interfacial evaporation. This study provides a theoretical foundation for understanding the interrelation between solar energy absorption, water transport, and salt diffusion in aerogel evaporators with hierarchical fiber‐pore architectures.

## Graphical abstract

The natural fiber@MXene‐engineered chitosan aerogel with hierarchically oriented channels is developed in this study. The photon‐entrapping networks cooperated with oriented channels to facilitate water transport and improve solar‐thermal/environmental energy synergy, endowing an evaporation rate up to 4.40 kg m^−2^ h^−1^ and efficient salt rejection performance with the composited aerogel evaporator, which is validated by both experiments and COMSOL simulations.

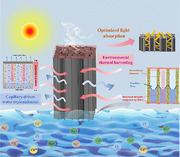

## 1 Introduction

Solar desalination techniques, which are low‐cost, sustainable, and environmentally friendly, have garnered significant attention from both industry and academia.^[^ ^1^, ^2^ ^]^ However, the low evaporation efficiency due to substantial heat loss severely limits their application. Recently, solar‐driven interface evaporation (SDIE) has been developed, offering enhanced evaporation efficiency due to localized heating at the evaporation interface.^[^ ^3^ ^]^ Among the various solar evaporators for SDIE, 3D evaporators have demonstrated unique advantages by harnessing additional energy from the environment, thus achieving higher evaporation rates than 2D evaporators.^[^ ^4^, ^5^ ^]^ Typically, carbon aerogels and polymer foam composites (PFCs) are promising candidates for 3D evaporators, owing to their excellent photothermal conversion performance and water transport capabilities.^[^ ^6^, ^7^ ^]^

Many biomass materials, such as wood and sunflowers, can be converted into carbon aerogels with abundant pores after calcination for SDIE.^[^ ^8^, ^9^, ^10^ ^]^ For instance, Wu et al. prepared a 3D solar evaporator from carbonized bamboo leaves with the aid of polyacrylamide, achieving an evaporation rate of 1.75 kg m^−2^ h^−1^ and an efficiency of 91.5% under one‐sun irradiation.^[^ ^11^ ^]^ Although biomass is abundant, its structural integrity and flexibility may weaken after calcination, limiting its widespread use in SDIE.^[^ ^12^, ^13^ ^]^ 2D light‐absorbing materials, such as graphene oxide and MXene, can be assembled to form 3D aerogels with a controllable porous structure for SDIE.^[^ ^14^, ^15^, ^16^ ^]^ The strong π−π and van der Waals interactions between these 2D materials provide flexibility and structural stability to the aerogels under external forces.^[^ ^17^, ^18^ ^]^ For PFCs, light‐absorbing nanomaterials (e.g., carbon nanotubes) are decorated onto the skeleton surface of polymer foams. However, the selective distribution of nanofillers on the skeleton surface, rather than a uniform mix with the polymer matrix, can lead to interfacial instability, especially during long‐term or cyclic SDIE, due to mismatched moduli and thermal conductivities between the rigid nanofillers and the soft polymer.^[^ ^19^ ^]^

As previously mentioned, 3D evaporators can harvest energy from the surrounding environment, enhancing the evaporation rate.^[^ ^20^, ^21^, ^22^ ^]^ The higher the evaporator is above the seawater surface, the more energy it can absorb. Therefore, it is crucial for 3D evaporators to efficiently pump seawater from the bulk to the evaporation surface at the highest possible level. Additionally, 3D evaporators must possess excellent mechanical properties and hence maintain structural stability and durability during long‐term SDIE.^[^ ^23^ ^]^

In this study, we develop MXene@kapok fiber (MKF)‐engineered chitosan aerogels with hierarchical channels for high‐efficiency solar‐driven steam generation. The MKF core‐shell configuration forms photon‐entrapping networks while suppressing MXene aggregation through spatial confinement, simultaneously enhancing the aerogel's mechanical properties and ensuring structural integrity. Both experimental results and COMSOL Multiphysics simulations demonstrate that hierarchical pore channels enhance water transport, improve solar‐thermal and environmental energy synergy, and facilitate the downward diffusion of concentrated ions from the evaporation surface, achieving an evaporation rate of 2.70 kg m⁻^2^ h⁻¹ with efficient salt rejection. The evaporator maintains stable performance in high‐salinity seawater, ensuring long‐term durability in solar‐driven interfacial evaporation. This work establishes a paradigm of bioinspired hierarchical engineering that concurrently regulates light absorption, water transport, and ion diffusion through multiscale architectural control, providing fundamental insights for the design of high‐performance solar evaporators.

## 2 Results and Discussion

**Figure 1:**
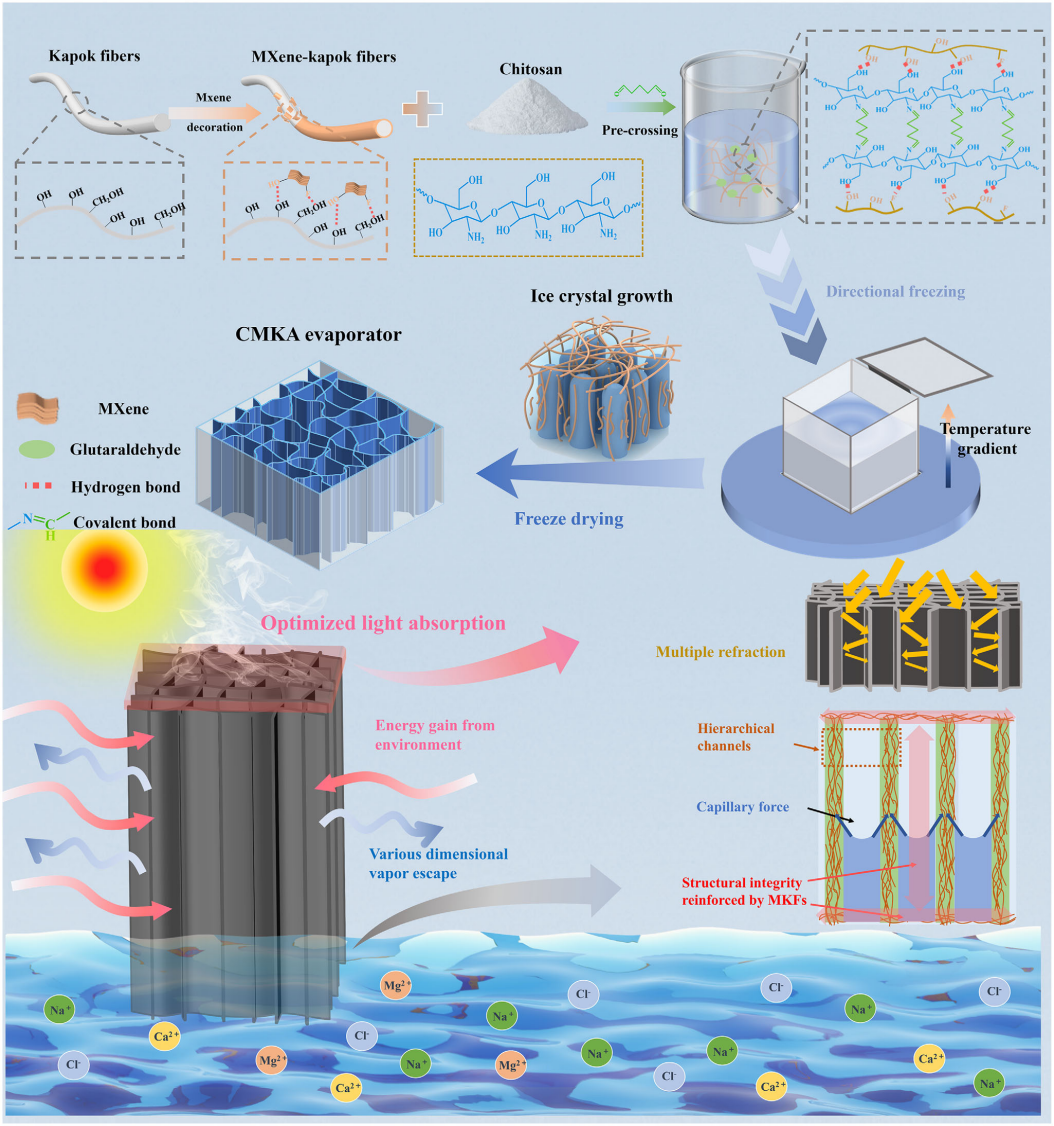
Schematic diagram of CMKA: preparation, structure, and application as solar evaporators.

This study develops a multiscale engineered chitosan/MXene‐kapok aerogel (CMKA) through a facile directional freezing assembly strategy, as illustrated in **Figure** **1**. The process innovatively integrates three pivotal strategies: 1) Hierarchical interface engineering, where chemically purified kapok fibers with oxygen‐enriched surfaces form interfacial hydrogen bonds with flexible MXene nanosheets,^[^ ^24^, ^25^ ^]^ creating MXene‐kapok fiber (MKF) heterostructures.^[^ ^26^ ^]^ These engineered interfaces not only prevent MXene aggregation but establish a hierarchical hydrogen‐bond architecture spanning molecular to micro scales through subsequent chitosan‐MKF interactions. 2) Anisotropic ice templating, where precisely controlled directional freezing induces ice growth along thermal gradients,^[^ ^27^ ^]^ simultaneously aligning macromolecular chains and MKFs in interstitial spaces while forming vertically oriented microchannels. This dual‐phase separation mechanism yields a wood‐inspired layered hydrogel architecture,^[^ ^28^ ^]^ which is converted into ultralight CMKA through freeze‐drying. The MXene localization strategy effectively circumvents nanosheet aggregation observed in conventional random distributions. 3) Synergistic multifunctional integration, where the aligned MKF network enables exceptional broadband light absorption at ultralow MXene content (≈1.00 wt.%) through continuous photon capture pathways,^[^ ^29^ ^]^ while the biomimetic microchannels achieve rapid water transport via optimized capillary pumping. The hydrogen‐bond reinforced interface design and 1D MKFs enhance mechanical resilience and brine stability through energy dissipation mechanisms, overcoming the inherent fragility of chitosan frameworks even in saline environments. This multiscale coordination strategy from molecular interface manipulation to macroscopic structural control establishes a new paradigm for high‐performance solar evaporation systems.

**Figure 2:**
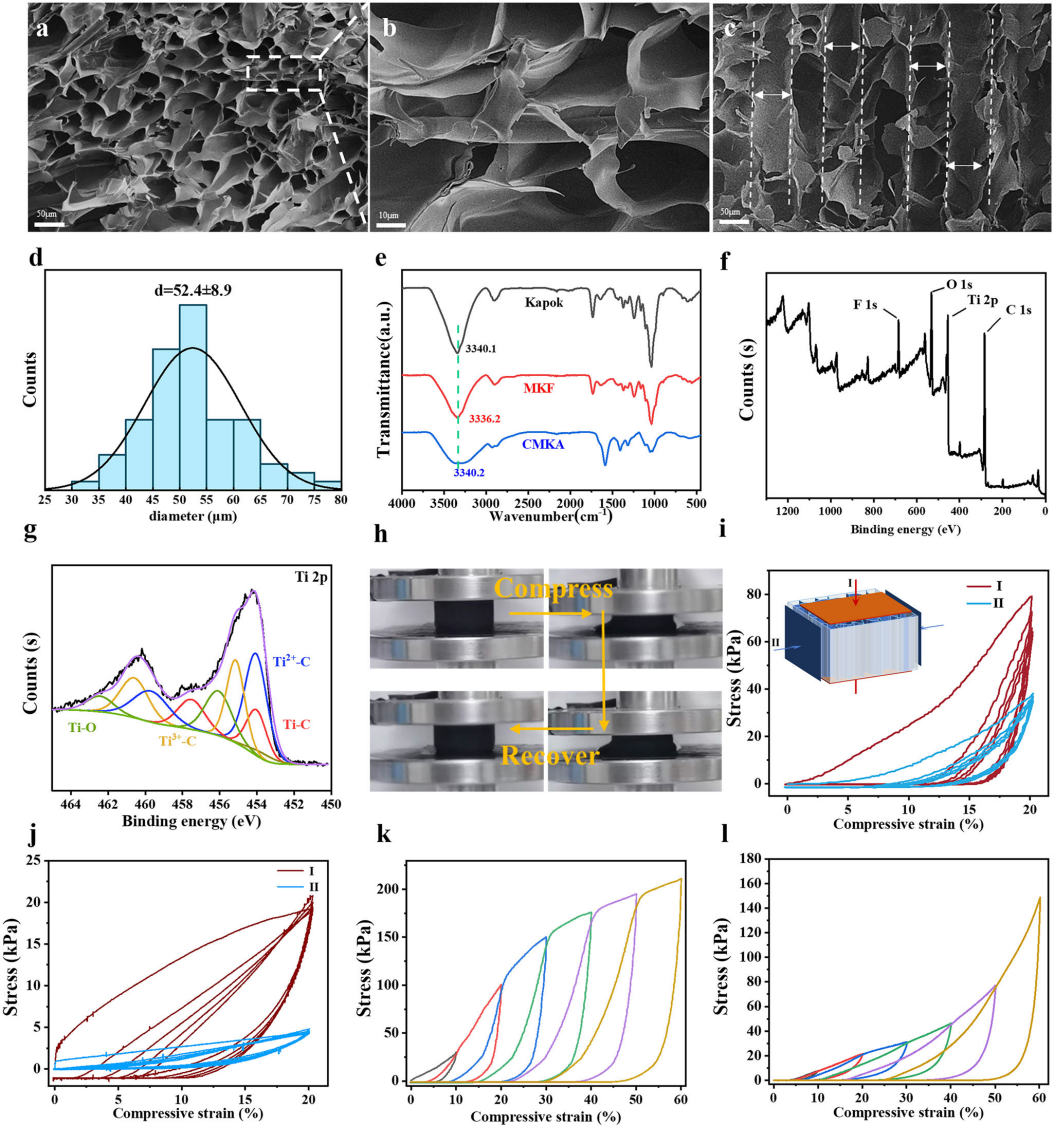
SEM images of CMKA: a,b) cross‐sectional views and c) sidewall view. d) Statistical distribution of pore sizes in CMKA. e) Infrared spectra of kapok fibers, MKFs, and CMKA. f,g) XPS spectrum and Ti 2p peak fitting of CMKA. h) Photographs showing the compression and rebound behavior of CMKA. i,j) Compressive stress–strain curves of (i) CMKA and (j) pure chitosan aerogel along two directions with 20% strain. Compressive stress along the vertical direction during loading and unloading cycles with a step‐increased strain of 10% for (k) CMKA and (l) pure chitosan aerogel.

**Figure** **2**a–c demonstrates a hierarchically ordered porous architecture with vertically aligned 50 µm channels, where MKFs either integrate into or bridge across the aerogel skeleton. Although the integration of MKFs heterostructures introduces localized structural heterogeneity, most of the hierarchically ordered porous channels retains their vertical orientation. The kapok fibers reduce the inherent brittleness of chitosan and increased the compressive strength and its fatigue resistance which are essential for long‐term operation in a high‐salt environment. Moreover, the biomimetic vascular design maintains hierarchical fluidic pathways for fast water convection within internal channels and enables high‐efficiency interfacial light absorption. The synergistic interplay between optimizes water transportation and photothermal conversion delivers a significant enhancement in solar‐driven interfacial evaporation performance. Hence, the synergistic evaporation‐enhancing effect of MKFs validates the rational trade‐off between localized structural compromises and the significant enhancement of core evaporation performance. The statistical distribution of pore size is shown in Figure 2d. The main pore diameters are concentrated ≈50 mm. There are a few secondary peaks in the data distribution peak, and these secondary peaks result from the structural irregularity introduced by the MKFs. Infrared spectroscopy analysis (Figure 2e) deciphers the interfacial chemistry evolution: The ─OH peak shift from 3340.1 cm⁻¹ (kapok) to 3336.2 cm⁻¹ (MKFs) confirms MXene‐kapok hydrogen bonding through F···H─O interactions,^[^ ^30^ ^]^ while its subsequent blueshift to 3340.3 cm⁻¹ in CMKA reveals chitosan‐MKF interfacial reorganization. XPS profiling (Figure 2f,g) verifies successful MXene incorporation while highlighting a critical innovation—the mild self‐assembly strategy reduces Ti─O species content to 17.0% versus 32.0% in conventional thermal‐stirring methods, demonstrating superior oxidation resistance. This interfacial stabilization mechanism stems from two factors: 1) MXene's directional assembly on kapok fibers minimizes nanosheet exposure to oxidative environments^[^ ^31^ ^]^; 2) Chitosan‐MKF hydrogen networks create protective molecular shielding. The combined effects enable exceptional MXene durability in saline conditions, addressing the chronic degradation challenge in photothermal materials. These structural insights establish a materials design paradigm where pore architecture engineering and molecular interface control mutually reinforce operational stability and evaporation performance.

We systematically investigate the multiscale mechanical engineering of CMKA through directional freeze‐drying, revealing three critical structural–performance relationships. First, as shown in Figures S1, S2 (Supporting Information), the aligned porous architecture enables low density while maintaining exceptional load‐bearing capacity (1 kg weight tolerance), a paradox resolved by its biomimetic anisotropic design. The vertical orientation of kapok fibers and chitosan frameworks creates a hierarchical reinforcement system, where macroporous channels (50 µm) serve as stress buffers and MKF‐chitosan hydrogen bonds act as molecular‐scale energy dissipators. To further investigate its mechanical properties, the compressive performance of CMKA in both the vertical (I) and horizontal (II) directions was analyzed. Figure 2i presents the compressive stress–strain curves over five cycles, showing pronounced hysteresis, especially during the first cycle, in both directions. Upon compression, the multiple hydrogen bonds between MKFs and chitosan break, leading to irreversible plastic deformation of the aerogel skeleton, resulting in energy loss during pressure release. CMKA demonstrates favorable modulus in the vertical direction, with the maximum compressive stress reaching ≈70.0 kPa after five loading and unloading cycles, compared to 30.0 kPa in the horizontal direction, highlighting the anisotropic nature of its mechanical properties.^[^ ^32^ ^]^ In contrast, the compressive stresses of pure chitosan aerogel in the vertical and horizontal directions are only ≈18.5 and 4.5 kPa, respectively. The plastic deformation of the chitosan skeleton is evident during compression cycles, as confirmed by the negative stress (≈−1.1 kPa) observed after compression unloading. This observation underscores the contribution of the oriented pore structure and kapok fibers to the enhanced mechanical properties of the aerogel. Figure 2k,l shows the compressive stress with step‐increased strain (10%) for dry CMKA and chitosan aerogel. The compressive stress of dry CMKA increases from 29.5 to 210.4 kPa as the compressive strain rises from 10% to 60%. In contrast, the compressive stress of pure chitosan aerogel increases from 7.8 to 148.7 kPa over the same strain range. CMKA demonstrates excellent resilience, recovering to its original shape after each cycle, demonstrating the reversible breaking and reconstruction mechanism of hydrogen bonds effectively suppresses permanent structural damage.^[^ ^33^ ^]^ Compared to pure chitosan aerogel, CMKA shows superior fatigue resistance and resilience. MKFs in the chitosan matrix act as a continuous phase filler, providing uniformly distributed hydrogen bonds. This enables more intermolecular fractures to occur through energy loss, improving the material's ability to resist compression. As shown in Figure S3 (Supporting Information), I‐CMKA undergoes compression tests, where its compressive stress increases from 33.8 to 173.8 kPa as the compressive strain increases from 10% to 60%. The oriented structure design optimizes the internal porosity and pore structure of the aerogel, effectively improving stress distribution under load. Since seawater is continuously pumped onto the evaporator surface during SDIE, the mechanical properties of CMKA after immersion in water for 0.5 min were also tested. As shown in Figure S4a (Supporting Information), the maximum compressive stress of CMKA in the wet state increases with strain, reaching 4.0, 12.1, 6.7, and 36.1 kPa at 10%, 30%, 50%, and 60% strain, respectively. Notably, the strength of CMKA in the wet state significantly decreases, which is attributed to the weakening of hydrogen bonds in the aerogel network caused by water adsorption.^[^ ^34^ ^]^ Although the CMKA skeleton is further stabilized by chemical crosslinking with small amounts of glutaraldehyde, the numerous hydrogen bonds between chitosan and MKFs are largely disrupted by water intrusion. However, despite the decrease in mechanical properties upon water exposure, CMKA still demonstrates satisfactory structural stability and resilience, making it suitable for seawater desalination applications. As shown in Figure S4b (Supporting Information), the chitosan aerogel skeleton collapses after water wetting, whereas the uniform packing distribution and continuous hydrogen bonds induced by MKFs help maintain the structural integrity of CMKA. Figure S4c (Supporting Information) presents photographs of wet CMKA during compression and recovery at strains of 20% and 60%. Clearly, the composite aerogel undergoes volume compression upon loading, expelling water stored in the pores and kapok fibers. Upon unloading, it reverts to its original size, and the extruded water is reabsorbed into the aerogel. The mechanical intelligence stems from synergistic design—MXene‐kapok interfaces maintain structural integrity during hydration, while aligned pores enable controlled water expulsion/reabsorption without permanent deformation. These innovations address the historical conflict between low density and mechanical robustness in solar evaporators, achieving simultaneous floatability, fatigue resistance, and brine durability—critical for practical seawater desalination.^[^ ^35^ ^]^

**Figure 3:**
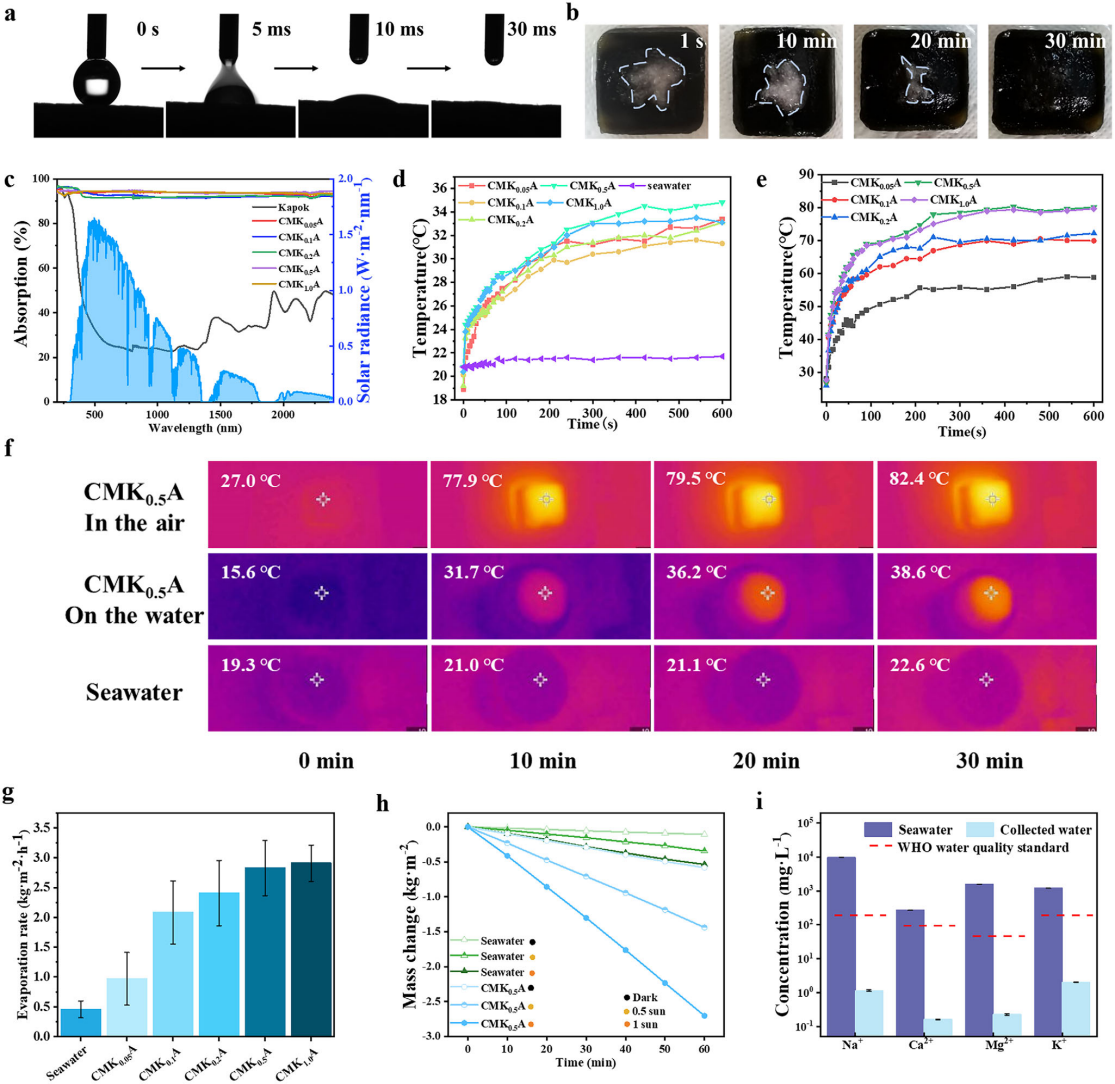
Water diffusion, photothermal conversion behavior, and SDIE performance of the composite aerogels. a) Photographs showing a water droplet diffusing on the surface of CMKA. b) Photograph of 3 g of sodium chloride crystals dissolving on the surface of wet CMKA. c) UV absorption spectra of kapok fibers and CMKA aerogels. d) Surface temperature variation of CMKAs exposed to one‐sun illumination in water. e) Surface temperature variation of CMKAs exposed to one‐sun illumination in the dry state. f) IR images showing surface temperature variations of seawater, CMKAs in air, and CMKAs in water. g) Diagram illustrating evaporation rates of CMKA evaporators with different MKF contents. h) Mass loss of seawater using CMKA under 0, 0.5, and 1 sun illumination. i) Ion concentrations of Na^+^, Ca_2_^+^, Mg_2_^+^, and K^+^ in original seawater and collected condensate.

Strong water transport capability is crucial for SDIE. The numerous oriented channels facilitate the transport of water from the bulk to the evaporator surface. The biomimetic microchannel architecture enables ultrafast hydraulic dynamics, with 5 µL water infiltration within 30 ms (**Figure** **3**a) through hierarchical capillary action spanning macroporous transport (50 µm channels) and nanoscale MXene‐kapok interfacial pumping. Simultaneously, these oriented fluidic networks achieve autonomous salt mitigation, dissolving 3 g NaCl in 30 min (Figure 3b) via continuous convective exchange between evaporation interfaces and bulk solution. The MKFs enhance the photothermal conversion performance of the aerogel. In Figure 3c, it can be seen that the original white kapok fibers exhibit only 17.5% light absorption in the visible range, while MKFs with a 0.05 wt.% MXene content achieves 92.7% absorption. Figure 3d,e shows the temperature variation of composite aerogels with different MKF contents under solar illumination in both water and air. Upon exposure, the absorbed light is converted to heat, causing the temperature to rise until it stabilizes at an equilibrium value. CMK_0.5_A achieves the highest equilibrium temperatures of 79.6 °C in air and 35.6 °C on the water surface after 5 min. The combination of high‐efficiency photothermal conversion and effective water transport enables rapid and stable interfacial evaporation. As shown in Figure 3f, CMK_0.5_A reaches ≈82.4 °C in 30 min under one‐sun illumination in air. In comparison, the water surface temperature rises by only 3.3 °C under the same sunlight exposure, while the surface temperature of CMK_0.5_A floating on the water increases by 23.0 °C. These results highlight the exceptional photothermal conversion and thermal localization capabilities of CMKA, which are crucial for efficient solar‐driven interfacial evaporation.

The desalination performance of CMKA‐based evaporators is systematically evaluated through evaporation rate measurements and salt rejection analysis. As shown in Figure 3g, the evaporation rates exhibit a positive correlation with MXene concentration, where CMK_1.0_A demonstrates a superior evaporation rate of 2.78 kg m^−2^ h^−1^ compared to 2.70 kg m^−2^ h^−1^ for CMK_0.5_A. This concentration‐dependent enhancement can be attributed to the improved broadband light absorption characteristics of MXene, which enhances photothermal conversion efficiency through intensified photon capture and electron‐phonon interactions. Despite the marginally lower performance, CMK_0.5_A is selected as the representative sample for standardized SDIE testing due to its optimal balance between material efficiency and evaporation performance. The evaporation dynamics are further investigated under controlled illumination conditions (Figure 3h). Notably, CMK_0.5_A demonstrates a baseline evaporation rate of 0.58 kg m^−2^ h^−1^ in dark conditions, substantially exceeding natural seawater evaporation (0.11 kg m^−2^ h^−1^). This remarkable dark evaporation enhancement originates from the hierarchical pore architecture, which simultaneously amplifies the effective evaporation surface area through fractal nano structuring and reduces vapor diffusion resistance via vertically aligned microchannels. Under solar irradiation, the evaporation rate escalates to 1.44 kg m⁻^2^ h⁻¹ (0.5 sun) and 2.70 kg m⁻^2^ h⁻¹ (1 sun), demonstrating efficient photothermal synergy. The linear evaporation kinetics suggest stable interfacial vapor generation with minimal thermal losses, indicative of optimized energy localization at the air‐liquid interface.

A critical challenge in solar desalination is salt accumulation, which typically degrades evaporation performance through light‐blocking crystalline deposits. The CMK_0.5_A evaporator addresses this limitation through its dual‐scale porosity: micron‐sized vertical channels enable rapid brine convection via capillary pumping, while nanoscale structures facilitate surface wetting through enhanced hydrophilicity. This structural design maintains continuous salt rejection without compromising evaporation stability. Post‐evaporation condensate analysis confirms exceptional desalination efficacy (Figure 3i). Ion concentrations demonstrate four‐order‐of‐magnitude reductions: Na^+^ (9693.4 → 1.49 mg L⁻¹), Ca^2^⁺ (270.0 → 0.16 mg L⁻¹), Mg^2^⁺ (1570.3 → 0.23 mg L⁻¹), and K⁺ (1200.4 → 2.00 mg·L⁻¹). These values not only surpass WHO drinking water standards but also validate the combined mechanisms of physical filtration through tortuous nanopores and ion adsorption via MXene's surface functional groups.

**Figure 4:**
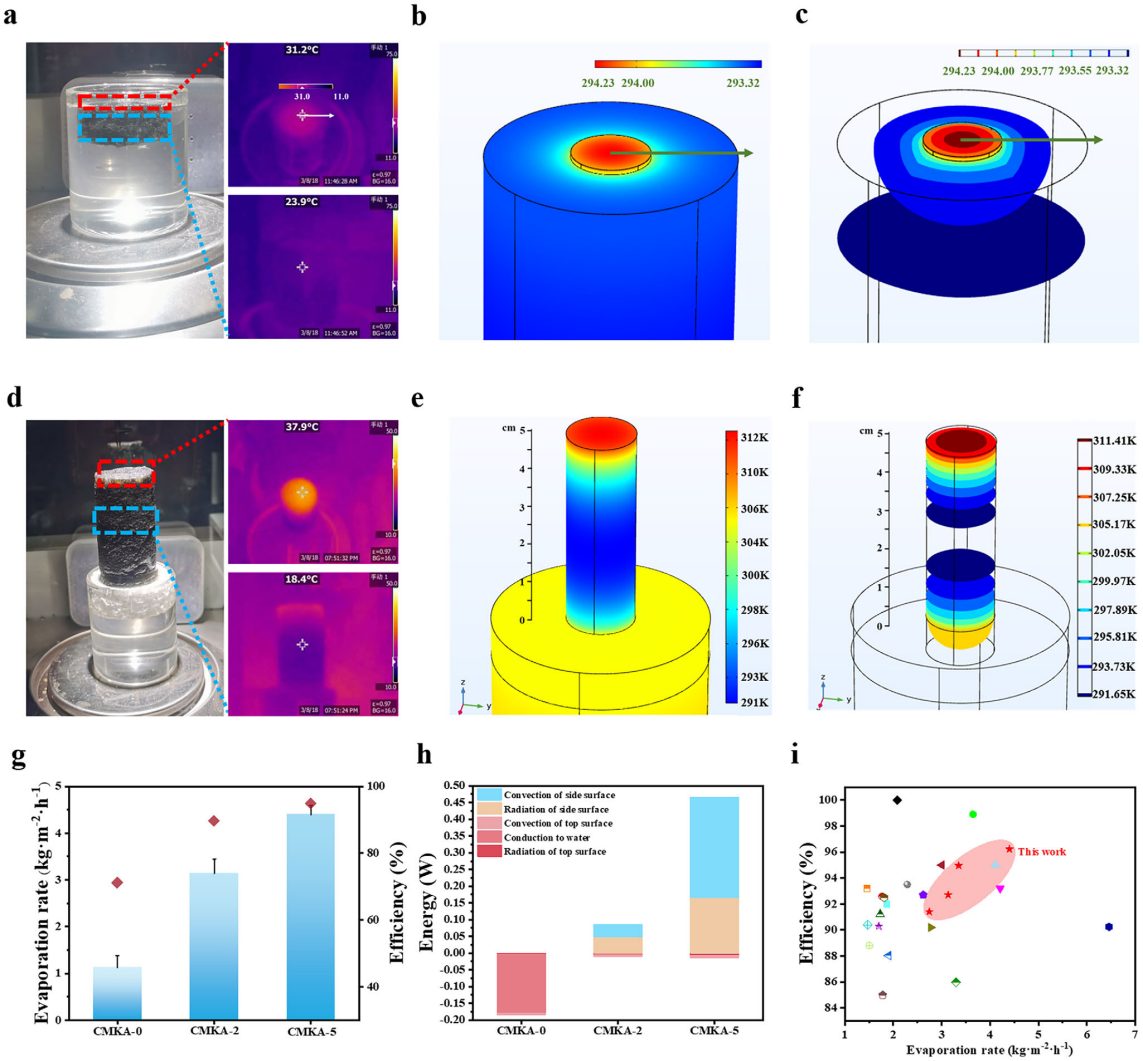
a) Photographs of CMKA‐0 during evaporation, along with infrared images of the evaporating surface and the water. b,c) Temperature distribution of CMKA‐0 after continuous evaporation for 6 h. (The upper surface of the cylinder and the hemispherical part represent the evaporator CMKA‐0 interface). d) Photographs of CMKA‐5 during evaporation, including infrared images of the evaporating surface and the middle of the composite aerogel. e,f) Temperature distribution of CMKA‐5 after continuous evaporation for 6 h. (The slender cylinder represents the CMKA‐5 evaporator, and the thicker cylinder below represents the bulk water). g) Evaporation rate and efficiency of CMKA‐based evaporators at different heights (the test materials are labeled as CMKA‐n, where n represents the height of the evaporating surface above the water). h) The energy distribution of the CMKA‐based evaporators used for SDIE in 1 sun. i) Comparison of evaporation rates and efficiencies of CMKA‐based evaporators with those of previously reported systems.

The height of the composite aerogel critically governs evaporation dynamics by modulating heat and mass transfer mechanisms. To systematically investigate this relationship, we analyze the evaporator height (denoted as CMKA‐n, where “n” represents the vertical distance between the evaporative surface and water interface) through thermodynamic and hydrodynamic perspectives. For the direct‐contact configuration (CMKA‐0), interfacial heat transfer exhibits dual adverse effects: rapid conductive heat dissipation from the photothermally interface to bulk water through the aerogel's directional porous network, compounded by convective heat exchange facilitated by the hydrophilic MKF‐enhanced water supply. These coupled mechanisms result in substantial thermal losses, as evidenced by the minimal temperature differential (ΔT = 7.3 °C) between the aerogel surface (31.2 °C) and bulk water (23.9 °C) under 1‐sun irradiation (**Figure** **4**a). The observed radial temperature gradient – decreasing from irradiation center to periphery – further confirms dominant lateral heat conduction along the water‐contacting plane. In contrast, elevating the evaporative surface (CMKA‐5) fundamentally alters the thermal transport regime. The 5 cm air gap establishes a localized heating zone by leveraging the aerogel's intrinsic low thermal conductivity, effectively decoupling the photothermal interface from bulk water. This spatial isolation minimizes conductive losses while expanding the evaporative surface area through sidewall exposure. The resultant vaporization enhancement creates a thermal gradient: upward capillary flow sustains evaporation‐driven cooling at the interface, while downward heat flux promotes secondary evaporation at sidewall interfaces. Crucially, the sidewall's sub‐ambient temperature enables passive energy harvesting from the environment, establishing a synergistic heat recuperation mechanism. This hierarchical thermal management combining localized heating, expanded evaporation boundaries, and environmental energy absorption, and demonstrates how vertical architecture engineering optimizes both thermal confinement and interfacial dynamics for enhanced evaporation performance.

Thermodynamic analysis of the composite aerogel's evaporative behavior, integrating COMSOL simulations and experimental validation, reveals critical insights into its thermal management mechanisms. As illustrated in Figure 4b,c, the simulated temperature gradient exhibits spherical dispersion from the evaporation surface to the ambient environment, reflecting isotropic heat dissipation characteristics that align closely with experimental observations. For the elevated CMKA‐5 configuration, the stabilized surface temperature of 37.9 °C under illumination (Figure 4d) underscores effective thermal localization, enabled by the aerogel's low thermal conductivity, which restricts conductive heat transfer to a shallow subsurface region (≈2 cm depth). The non‐monotonic sidewall temperature profile, i.e., initially decreasing from the top before rising slightly toward the base, is attributed to competing thermodynamic processes: evaporative cooling dominates the upper regions, extracting latent heat and creating a mid‐height temperature minimum (18.4 °C), while gradual heat‐absorption from bulk water and reduced evaporation rates at lower elevations drive the subsequent temperature recovery. Cross‐sectional thermal mapping (Figure 4e,f) further quantifies this vertical gradient, with the evaporation interface maintaining 311.27 K (38.27 °C), followed by a progressive decline to 291.65 K (18.65 °C) at the mid‐sidewall, and the simulation results are consistent with experimental infrared measurements. This stratified thermal architecture highlights two synergistic mechanisms: 1) surface‐localized photothermal conversion minimizes parasitic losses to bulk water, while 2) sidewall evaporative cooling establishes a sub‐ambient thermal sink that passively harvests environmental energy. The remarkable congruence between simulated and experimental profiles validates the model's predictive capability for optimizing aerogel geometries, particularly in balancing vertical thermal confinement with lateral environmental energy recuperation.

The evaporation performance metrics and energy partitioning analysis demonstrate a pronounced height‐dependent optimization mechanism in the CMKA architecture. As shown in Figure 4g, the evaporation rate escalates from 1.13 kg m⁻^2^ h⁻¹ for the submerged CMKA‐0 to 3.31 kg m^−2^ h^−1^ (CMKA‐2) and 4.40 kg m^−2^ h^−1^ (CMKA‐5), directly correlating with the exposed sidewall area above the water interface. To evaluate the evaporation efficiency of the CMKA‐based evaporators, we divided the total energy input into two components: energy converted by solar illumination and heat drawn from the surrounding environment. The energy from photothermal conversion at the interface primarily drives water evaporation, though a portion is lost to the environment via conduction, convection, and radiation. Therefore, combined with Equations (S6–S11) (Supporting Information), the convective and radiative heat at the top is regarded as the heat lost by the evaporator system. When the evaporation interface is located above the water surface, the increased evaporation area enhances evaporation along the side walls, leading to a further reduction in the side wall temperature. Based on the experimental and simulation results mentioned above, the heat conduction loss through the side walls can be considered negligible when the side wall temperature is lower than the bulk water temperature.^[^ ^21^ ^]^ The sidewall surface temperature of CMKA remains below ambient temperature, enabling effective heat absorption from the surrounding environment through combined convective and radiative transfer mechanisms. This thermodynamic characteristic necessitates the inclusion of ambient energy contributions when evaluating evaporation efficiency. When analyzing the evaporation efficiency of CMKA‐2 and CMKA‐5, we considered that heat transferred downward from the evaporating surface contributes to the evaporation process. From an energy balance perspective, convective and radiative losses at the upper surface were classified as system energy dissipation, while the corresponding heat transfer phenomena occurring at the sidewalls were treated as environmental energy replenishment to the system. As shown in Figure 4h, the light utilization efficiency of CMKA‐0 is 73.6%, with radiative heat loss accounting for 3.4%, conductive heat loss at 17.5%, and convective heat loss at 5.5%. In contrast, CMKA‐2 and CMKA‐5 exhibit evaporation efficiencies of 89.6% and 94.8%, respectively, with environmental heat contributions accounting for 15.4% and 37.4% of the total vaporization energy, a direct consequence of sidewall‐mediated energy recuperation. A comparison with recently reported evaporators (Figure 4i) demonstrates that our composite aerogel evaporators show the unique advantages in both the evaporation rates and efficiencies. The close agreement between modeled energy flows and experimental data validates the hierarchical thermal management strategy, where geometric elevation transforms conventional interfacial evaporation into a volumetrically optimized, environment‐coupled process.

**Figure 5:**
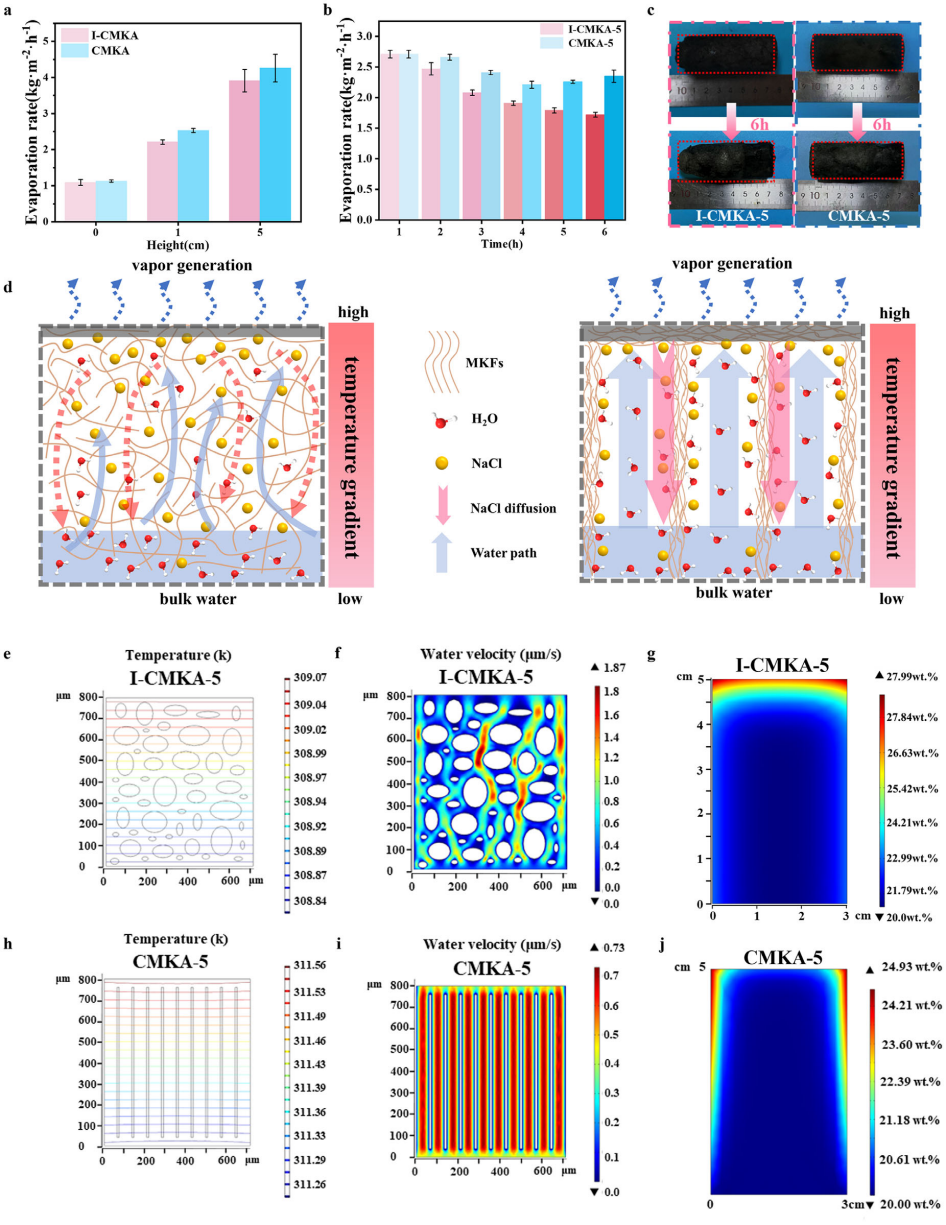
a) Evaporation rates of evaporators based on CMKA and I‐CMKA at different heights (0, 1, and 5 cm represent the height of the evaporating surface above the water surface). b) The per‐hour evaporation rate of evaporators based on CMKA‐5 and I‐CMKA‐5, continuously evaporating for 6 h at 20 wt.% seawater. c) Images of CMKA‐5 and I‐CMKA‐5 before and after 6 h of irradiation in 20 wt.% brine, respectively. d) Schematics of the water flow path and salt resistance of I‐CMKA‐5 and CMKA‐5. e,f) Simulated temperature and water velocity distributions in the internal channels of I‐CMKA‐5 during evaporation. h,i) Simulated temperature and water velocity distributions in the internal channels of CMKA‐5 during evaporation. g,j) Simulated salt diffusion paths in I‐CMKA‐5 and CMKA‐5, respectively. (The bottom of the evaporator is in direct contact with the water). Note: The simulation was performed using COMSOL Multiphysics 5.5.

The effect of exposure height on the evaporation rate of both isotropic and anisotropic evaporators is assessed (**Figure** **5**a). As height increases, the exposed surface area expands, allowing greater energy absorption from the surrounding air and enhancing evaporation. Consequently, both CMKA and I‐CMKA evaporators exhibit increased evaporation rates in 3.5 wt.% salinity seawater (Figure 5a). At each height, CMKA outperforms I‐CMKA due to its directional water channels, which facilitate efficient water transport from the bottom to the evaporation interface, promoting enhanced evaporation. However, the influence of exposure height diminishes over time, as the gravitational potential energy required for water transport becomes more significant at greater heights. Based on these findings, a fixed height of 5 cm is selected for subsequent experiments.

To evaluate water transport and salt resistance under extreme conditions, continuous evaporation of 20 wt.% salinity seawater is conducted over 6 h (Figure 5b). CMKA‐5 maintains a stable evaporation rate of ≈2.43 kg m^−2^ h^−1^ throughout the experiment. In contrast, the evaporation rate of I‐CMKA‐5 gradually declines from 2.71 kg m^−2^ h^−1^ to 2.08 kg m^−2^ h^−1^ and further to 1.72 kg m^−2^ h^−1^ as evaporation progresses. The optimized water transport pathways in CMKA support sustained solar steam generation. Furthermore, CMKA‐5 demonstrates superior compression resistance, preventing structural deformation and contraction in brine. This mechanical integrity ensures consistent evaporation performance without structural compromise. As shown in Figure 5c, CMKA‐5 largely retains its initial structure after 6 h of continuous evaporation in 20 wt.% seawater, exhibiting only minor shrinkage (4.04%). In contrast, I‐CMKA‐5, which lacks efficient water transport due to discontinuous internal channels, experiences severe shrinkage at the top, leading to an overall volume reduction of 20.18%. Elemental analysis of sectioned samples after 6 h of continuous evaporation (Figure S6, Supporting Information) reveals that CMKA‐5 develops only a thin salt layer, formed by residual salts crystallizing upon drying. In contrast, I‐CMKA‐5 exhibits substantial salt accumulation, blocking its internal channels. This obstruction impairs water transport and induces further structural contraction, compromising performance.

Figure 5d schematically illustrates the mechanistic advantages of CMKA‐5′s oriented architecture in hypersaline environments, compared to the isotropic structure of I‐CMKA‐5. While both systems exhibit comparable initial evaporation rates due to similar surface thermodynamics, their structural divergence fundamentally alters hydraulic and ionic transport dynamics. In I‐CMKA‐5, stochastic fiber distribution in a nonoriented porous structure creates tortuous capillary pathways (Figure 5d) that impede smooth flow, exacerbating salt accumulation at the evaporation interface. The thermally induced concentration gradient drives downward ion diffusion, but the disordered polymer network acts as a percolation barrier, enabling rapid supersaturation and interfacial salt nucleation, which ultimately degrades the interfacial evaporation performance.^[^ ^36^, ^37^ ^]^ In contrast, CMKA‐5′s vertically aligned channels establish low‐resistance hydraulic pathways, enabling synergistic transport mechanisms: 1) upward capillary flow sustains evaporation‐driven water replenishment, while 2) downward Marangoni convection (driven by salinity gradients) continuously dilutes interfacial brine. This bidirectional flux maintains sub‐saturation salinity at the photothermal layer, preventing crystalline blockage. The effectiveness of capillary water absorption to the top surface of the composite aerogel through the oriented channels has been demonstrated (Figure S7, Supporting Information). In the CMKA sample, water is transported upward over a distance of 4 cm in ≈248 s, while in the I‐CMKA sample, the same distance takes 308 s. These results highlight that the regular, directional channels in the CMKA structure reduce the resistance to water transport, allowing for faster upward movement of water. The upward capillary force thus facilitates rapid replenishment of water in the system.^[^ ^38^ ^]^

Multiphysics simulations quantitatively demonstrate how CMKA‐5′s directional architecture outperforms isotropic designs in hydraulic efficiency, thermal localization, and salt mitigation (Figure 5e−g). The vertically aligned channels in CMKA‐5 enhance photothermal conversion efficiency compared to I‐CMKA‐5, achieving a steeper vertical temperature gradient that intensifies interfacial vaporization while minimizing lateral heat dissipation (Figure 5e,h). Additionally, under conditions where pore sizes are similar, the water pressure simulation in Figure S8 (Supporting Information) reveals that both aerogel evaporators generate negative relative water pressure at the top, ensuring a continuous upward water supply from the bottom. The negative pressure generated by the direction of water flow in a water supply channel is correlated with its flow velocity. When water is transported from bottom to top, the driving force needs to overcome both gravity and negative pressure to ensure stable water transport through the voids. Hydraulic analysis reveals that CMKA‐5′s low‐tortuosity pathways reduce the flow resistance, requiring only 0.73 µm s^−1^ velocity and 7.35 Pa negative pressure to sustain capillary‐driven water supply—a 61% lower velocity demand than I‐CMKA‐5 (1.87 µm s^−1^ at 7.34 Pa), as shown from the simulated results in Figure 5f,i. This hydraulic advantage stems from minimized gravitational counterflow, where streamlined channels counteract gravity through optimized Laplace pressure gradients.

Numerical modeling of hypersaline transport dynamics (CMKA in a 20 wt.% high‐salinity water solution for 6 h of continuous evaporation) through COMSOL simulations elucidates the structural determinants of CMKA evaporators’ exceptional salt rejection. The fluid transport mechanism critically governs salt management: CMKA's directional flow sustains a self‐regulating salinity ceiling (23.54 wt.% for CMKA‐1, 24.69 wt.% for CMKA‐3 and 24.93 wt.% for CMKA‐5, all below NaCl saturation) by coupling rapid upward replenishment with downward ion dispersion via Marangoni flows. In contrast, I‐CMKA‐5′s disordered pores permit localized supersaturation (27.99 wt.%), exceeding the percolation threshold for salt nucleation (Figure 5g,j). This divergence arises from CMKA‐5′s synergistic transport—capillary forces maintain hydration fronts that dilute interfacial brine, while aligned channels enhance ionic diffusivity, redistributing salts into bulk water. The results on salt rejection align with the experimental findings (Figure S9, Supporting Information). These results validate that oriented porosity not only optimizes hydraulic permeability but also decouples heat‐mass transfer processes, directing thermal energy toward vaporization while channeling ionic species away from interfaces. This structural design enables CMKA evaporators to overcome the ‘self‐clogging’ limitations of conventional evaporators, where flow resistance, thermal losses, and salt occlusion severely degrade interfacial evaporation performance. By leveraging geometry‐engineered transport synergy, CMKA achieves sustainable desalination.

**Figure 6:**
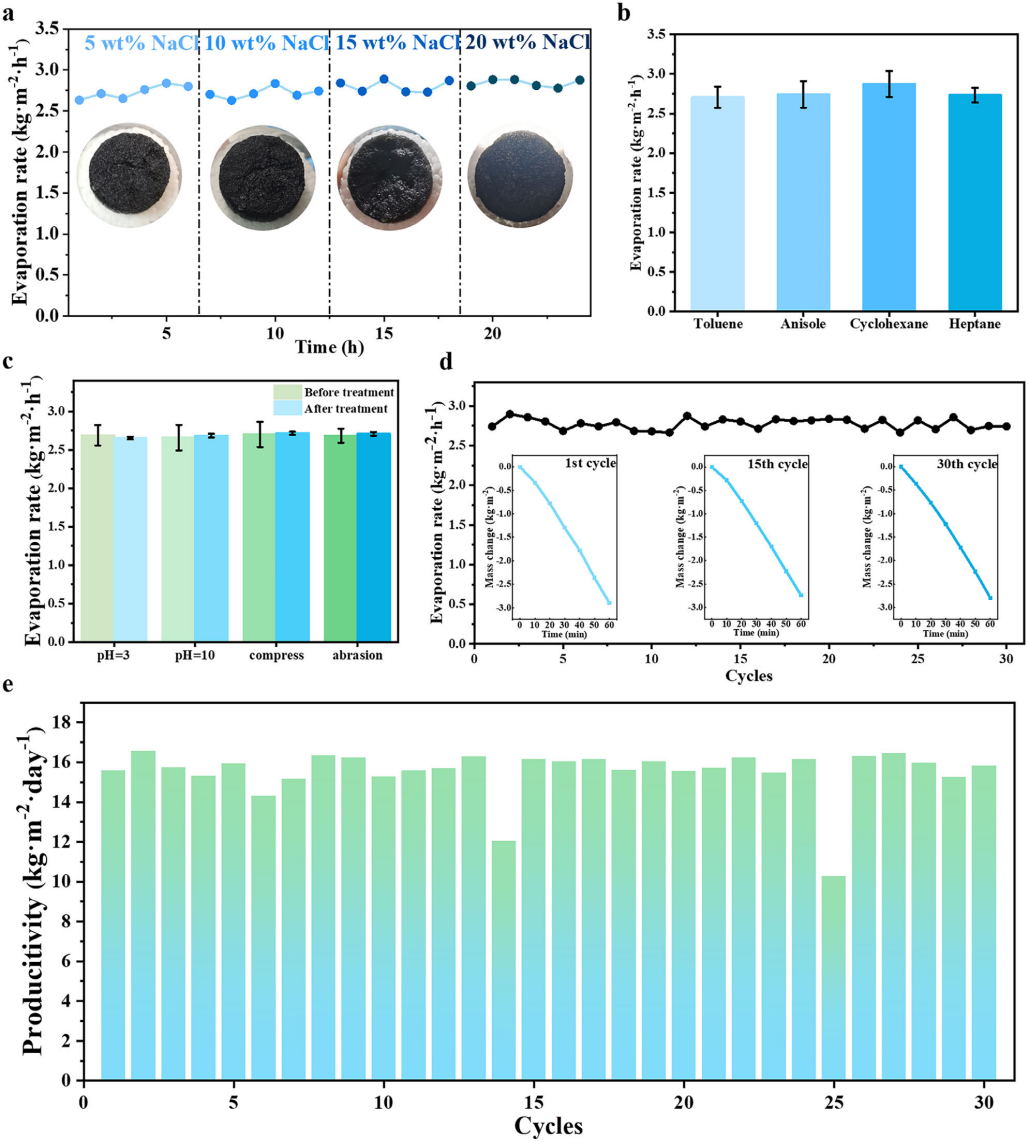
a) Evaporation rates of saline water with NaCl concentrations of 5, 10, 15, and 20 wt.%. Insets: photographs of the CMKA‐based evaporator surface after each evaporation cycle. b) Evaporation rate of the CMKA‐based evaporator in different oil‐in‐water emulsions. c) Evaporation rate under complex environmental conditions. d) Evaporation performance over 30 cycles. Insets: mass loss of saline water over time for the 1st, 15th, and 30th cycles. e) Daily water volume produced by the CMKA‐based evaporator under actual outdoor sunlight.

Superior salt rejection capability governs the long‐term operational viability of solar evaporators, as interfacial salt nucleation critically degrades photothermal efficiency through light scattering and hydraulic channel occlusion.^[^ ^39^, ^40^, ^41^ ^]^ Laboratory‐scale validation (Figure S10, Supporting Information) and hypersaline testing (**Figure** **6**a) demonstrate CMKA_‐0.5_A‐1′s exceptional durability, maintaining a stable evaporation rate of 2.70 kg m^−2^ h^−1^ across multiple cycles in brines up to 20 wt.% NaCl. The CMKA evaporator demonstrates satisfactory operational robustness in complex aquatic environments, overcoming critical challenges posed by hydrocarbon pollutants, corrosive conditions, and mechanical fatigue through integrated material and structural engineering. Its superhydrophilic pore architecture thermodynamically exclude nonpolar contaminants such as cyclohexane droplets in oil‐water emulsions while sustaining unidirectional capillary‐driven transport. This molecular discrimination enables stable evaporation rates of 2.70 kg m^−2^ h^−1^ in hydrocarbon‐laden brines (Figure 6b), matching pure seawater performance. Optical characterization (Figure S11, Supporting Information) confirms the condensate's oil‐free composition, validating molecular sieving through surface energy optimization.

To evaluate the material stability, the composite aerogel's evaporation rate was tested after exposure to solutions with pH 3 and 10 for 24 h, 100 compression cycles (with 30% deformation), and surface abrasion with 200‐mesh sandpaper. The results, presented in Figure 6c, show that the evaporation rate remained consistent before and after these treatments, indicating the structural integrity of the composite aerogel. To further assess the stability of the CMKA‐based evaporator, 30 evaporation cycles were performed, with results shown in Figure 6d. During these cycles, the evaporation rate fluctuated ≈2.70 kg m^−2^ h^−1^, and the mass loss curves for the 1st, 15th, and 30th cycles were similar, demonstrating the evaporator's stability over extended use. These results confirm that CMKA maintains its photothermal conversion properties and interfacial stability, highlighting its suitability for solar‐driven interfacial evaporation in complex environments. Outdoor deployment metrics (Figure 6e) validated practical viability, with daily freshwater yields of 15.7 L·m^−2^ under natural sunlight, sufficient for four adults’ needs. The condensate's electrical resistivity (1.394 MΩ) matched DI water standards (Figure S12, Supporting Information), confirming complete salt and contaminant rejection. These results establish a technological paradigm where hierarchical architecture combining directional hydraulics, contaminant‐resistant surfaces, and mechanically robust nanocomposites enables sustainable solar desalination in real‐world polluted or hypersaline environments, transcending the limitations of conventional interfacial evaporators.

## 3 Conclusion

We present a hierarchically engineered chitosan aerogel reinforced with natural fiber@MXene through unidirectional freeze‐casting, featuring multiscale vertically aligned channels that synergistically enhance photothermal conversion, hydraulic dynamics, and thermal management. The MKFs act as broadband solar absorbers (92.7% absorption across 250–2500 nm), enabling efficient interfacial heating (surface temperatures ≈38 °C under one sun), while the aligned microchannels minimize hydraulic tortuosity, achieving rapid capillary‐driven water transport that suppresses salt nucleation. The CMK_0.5_A‐5 demonstrates an exceptional evaporation rate of 4.40 kg m^−2^ h^−1^ and 94.8% efficiency by leveraging dual energy harvesting: direct solar‐thermal conversion coupled with environmental heat absorption through sub‐ambient sidewall cooling (18.4 °C mid‐height). COMSOL simulations corroborate experimental salt distribution profiles, revealing interfacial salinity <25 wt.% in 20% brine. The superhydrophilic evaporator selectively excludes hydrocarbons, maintaining stable evaporation in oil emulsions (2.70 kg m⁻^2^ h⁻¹) and corrosive media (pH 3–10). Outdoor trials yielded 15.7 L m^−2^ day^−1^ of high‐purity condensate (1.394 MΩ resistivity), validating CMKA as a robust, scalable solution for hypersaline and polluted water remediation through architecture‐governed heat‐mass synergy.

## 4 Experimental Section

### Materials

MAX powders were purchased from Laizhou Kai Kai Ceramic Materials Co., Ltd. Lithium bifluoride (LiF), hydrochloric acid (HCl), and chitosan were obtained from Sinopharm Chemical Reagent Co., Ltd. All chemicals and reagents were of analytical‐grade purity and used as received. Kapok fibers with a hollow structure (≈10 µm in diameter) were sourced from Yunnan Ming Yang and degreased by boiling in an alkaline solution before use, as described in the previous report.^[^ ^24^, ^42^ ^]^

### Fabrication of Chitosan/MXene/Kapok Fibers Composite Aerogel (CMKA)

MXene nanosheets were obtained via the HF etching method.^[^ ^43^ ^]^ The MXene‐decorated kapok fibers (MKFs) were fabricated through a solution‐based assembly strategy. Initially, degreased kapok fibers were combined with MXene nanosheets in a 10:1 mass ratio within 200 mL of deionized water (DI) under high‐speed homogenizer treatment. This intensive mixing process facilitated the uniform assembly of MXene nanosheets onto the kapok fiber surfaces through hydrogen bond interactions. Subsequently, the resulting homogeneous suspension underwent a freeze‐drying process (−80 °C, 0.1 mbar, 48 h) to remove the aqueous medium while maintaining the porous structure, ultimately yielding the MKFs composite with stable MXene‐kapok interfacial integration.^[^ ^43^ ^]^ Next, 5 g of chitosan powder was added to 95 g of DI water in a beaker, and a 5 wt.% chitosan solution was obtained by magnetic stirring at 40 °C. MKFs were dispersed in the chitosan solution and mechanically stirred in an ice water bath for 4 h to prepare a uniformly dispersed chitosan/MKFs slurry. To cross‐link the slurry, 5 mL of glutaraldehyde solution (2 wt.%) was added, and the mixture was stirred at low speed. The slurry was then poured into a mold and cross‐linked at room temperature for 30 min to form the stable chitosan/MKFs composite hydrogel. The hydrogel was subsequently placed on top of a copper cylinder, with the bottom immersed in liquid nitrogen (≈196 °C), for directional freezing. Finally, the directionally frozen samples were vacuum‐dried to form chitosan/MKFs composite aerogels. The samples were named CMKxA, where x denotes the mass ratio of MKFs to chitosan solution. The samples used for characterization and mechanical property testing were CMK_0.5_A unless otherwise specified. In the comparison experiment, I‐CMK_0.5_A (the isotropic aerogel samples) was used to study the effect of the composite aerogel structure on evaporation performance, where the mass ratio of MKFs to chitosan solution was 0.5.

### Characterization

The surface and sectional morphologies of the samples were observed using scanning electron microscopy (SEM, Zeiss Supra55, Germany) at an accelerated voltage of 5 kV. The elemental distribution of the composite aerogels was analyzed using energy dispersive spectroscopy (EDS). The aerogel samples were fractured in liquid nitrogen along both the parallel and vertical directions, then spray‐coated with a thin layer of gold for SEM observation. Functional groups in the samples were characterized using a micro‐infrared spectrometer (Cary 610/670, Varian Co. Ltd., U.S.A.). X‐ray photoelectron spectroscopy (XPS, ESCALAB 250Xi) was employed to determine the chemical composition of the aerogels. Optical reflectance and transmittance of the Kapok fibers, MKFs, and CMKAs were measured using a UV/VIS/NIR spectrometer (PerkinElmer, Lambda 750S), with a wavelength range of 200 to 2500 nm.

### Solar‐Driven Interfacial Evaporation Measurements

During the SDIE tests, polystyrene (PS) foam was chosen as the insulation layer, featuring a centrally located square hole measuring 1.5 × 1.5 cm^2^. The CMKA‐based evaporator was placed within the PS foam hole to serve as the evaporation device. The setup was positioned in a breaker and illuminated by a Xenon lamp (PLS‐SXE300+/UV, Prefect Light, Beijing, China), which functioned as a solar simulator. The mass change during the interfacial evaporation process was measured using a digital balance (PTX‐FA210, Fujian, China). Additionally, the temperature of both the light‐absorbing surface and evaporation side was recorded using an infrared (IR) thermal imager (TiS10, Fluke) to evaluate light absorption and thermal management performance. The ion concentration before and after the desalination process was quantified using inductively coupled plasma‐optical emission spectrometry (ICP‐OES, Optima 7300 DV, PerkinElmer). The simulated interfacial evaporation tests were performed at room temperature (≈30 °C) and air humidity of ≈40%. The relevant formula for the SDIE experiments is as follows:

(1) $$v=\frac{\Delta m}{s\;\times \;t}\;$$

where *∆m* denotes the change in mass of seawater (in kg), *s* represents the irradiated area (in m^2^, with a value of 10^−4^ m^2^ used in the evaporation tests), and *t* indicates the duration of evaporation (in hours). In both laboratory and outdoor evaporation tests, the height of the evaporator above the water surface was consistently set at 1 cm. The sample CMK_0.5_A‐1 was used as the standard for the SDIE evaporation tests.

## Conflict of Interest

The authors declare no conflict of interest.

## Supporting information



Supporting Information

## Data Availability

The data that support the findings of this study are available from the corresponding author upon reasonable request.

## References

[1] C. Ge , D. Xu , H. Du , Z. Chen , J. Chen , Z. Shen , W. Xu , Q. Zhang , J. Fang , Adv. Fiber Mater 2023, 5, 791.

[2] Y. Dong , Y. Lin , C. Du , C. Zhou , S. Yang , Coll. Surf. A 2022, 643, 128755.

[3] Q. Su , J. Yan , W. Xiao , Y. Liu , L. Li , J. Wang , X. Huang , H. Xue , J. Gao , Chem. Eng. J. 2024, 490, 151814.

[4] J. Ma , Y. Xu , Y. Xu , L. An , W. Wang , Environ. Sci. Technol. 2023, 57, 10652. 37458075 10.1021/acs.est.3c02454

[5] S. Meng , C.‐Y. Tang , J. Yang , M.‐B. Yang , W. Yang , Adv. Sci. 2022, 9, 2204187. 10.1002/advs.202204187 PMC9685475 36216571

[6] W.‐M. Zhang , J. Yan , Q. Su , J. Han , J.‐f. Gao , J. Colloid Interface Sci. 2022, 612, 66. 34974259 10.1016/j.jcis.2021.12.093

[7] G. Hu , H. Liu , K. Liu , H. Wang , X. Wen , L. Liu , Y. She , L. Feng , R. Niu , J. Gong , Adv. Funct. Mater. 2025, 2423781.

[8] S. Zhang , M. Li , C. Jiang , D. Zhu , Z. Zhang , Adv. Sci. 2024, 11, 2308665. 10.1002/advs.202308665 PMC11077647 38342614

[9] Y. Geng , W. Sun , P. Ying , Y. Zheng , J. Ding , K. Sun , L. Li , M. Li , Adv. Funct. Mater. 2021, 31, 2170020.

[10] X. Su , D. Hao , M. Sun , T. Wei , D. Xu , X. Ai , X. Guo , T. Zhao , L. Jiang , Adv. Funct. Mater. 2022, 32, 2108135.

[11] Y. Wu , R. Kong , C. Ma , L. Li , Y. Zheng , Y. Lu , L. Liang , Y. Pang , Q. Wu , Z. Shen , H. Chen , Energy Environ. Mater. 2022, 5, 1323.

[12] K. Fang , C. Du , J. Zhang , C. Zhou , S. Yang , J. Membr. Sci 2022, 663, 121037.

[13] J. Xu , R. Cui , C. Zhou , S. Yang , Desalination 2024, 573, 117204.

[14] L. Zhao , C. Du , C. Zhou , S. Sun , Y. Jia , J. Yuan , G. Song , X. Zhou , Q. Zhao , S. Yang , ACS Sustain Chem Eng 2020, 8, 4362.

[15] W. Xiao , J. Yan , S. Gao , X. Huang , J. Luo , L. Wang , S. Zhang , Z. Wu , X. Lai , J. Gao , Desalination 2022, 524, 115475.

[16] Y. Ma , Y. Hu , N. Li , Y. Wang , J. Yu , Z. Hu , Adv. Funct. Mater. 2025, 2422725.

[17] S. Wang , L. Yu , S. Wang , L. Zhang , L. Chen , X. Xu , Z. Song , H. Liu , C. Chen , Nat. Commun. 2022, 13, 3408. 35729107 10.1038/s41467-022-30224-8 PMC9213515

[18] X. Jing , L. Chen , Y. Li , H. Yin , J. Chen , M. Su , F. Liu , T. Abdiryim , F. Xu , J. You , X. Liu , Small 2024, 20, 2405587. 10.1002/smll.202405587 39350451

[19] J. Han , W. Xing , J. Yan , J. Wen , Y. Liu , Y. Wang , Z. Wu , L. Tang , J. Gao , Adv. Fiber Mater. 2022, 4, 1233.

[20] J. Yan , T. Cui , Q. Su , H. Wu , W. Xiao , L. Ye , S. Hou , H. Xue , Y. Shi , L. Tang , P. Song , J. Gao , Adv. Sci. 2024, 11, 2407295. 10.1002/advs.202407295 PMC11538639 39234809

[21] J. Yan , Q. Wu , J. Wang , W. Xiao , G. Zhang , H. Xue , J. Gao , J. Colloid Interface Sci. 2023, 641, 1033. 36996682 10.1016/j.jcis.2023.03.114

[22] X. Liu , F. Chen , Y. Li , H. Jiang , D. D. Mishra , F. Yu , Z. Chen , C. Hu , Y. Chen , L. Qu , W. Zheng , Adv. Mater. 2022, 34, 2203137. 10.1002/adma.202203137 35839320

[23] J. Wang , W. Wang , L. Feng , J. Yang , W. Li , J. Shi , T. Lei , C. Wang , Sol. Energy Mater. Sol. Cells 2021, 231, 111329.

[24] L. Li , Q. Su , W. Xiao , J. Yan , H. Wu , J. Wang , Z. Liu , H. Li , H. Xue , L. Wang , Y. Shi , L. Tang , J. Gao , Composites, Part B 2025, 296, 112235.

[25] W. Xiao , H. Wu , Q. Su , J. Yan , L. Tang , X. Huang , L. Lu , W. Gu , P. Song , J. Gao , Chem. Eng. J. 2025, 508, 161074.

[26] W. Xiao , Y. Chen , G. Pan , J. Yan , J. Zhang , J. Gao , Adv. Fiber Mater. 2023, 5, 2040.

[27] Y. Wang , D. Wu , J. Mater. Chem. A 2024, 12, 4099.

[28] Y. Ko , S. Lee , J. Jang , G. Kwon , K. Lee , Y. Jeon , A. Lee , T. Park , J. Kim , J. You , Adv. Funct. Mater. 2025, 35, 2414576.

[29] J. Y. Wu , D. Liu , Y. X. Sun , B. K. Wei , K. Dai , Y. Q. Sun , F. Zhang , C. B. Li , J. Xue , Z. F. Zhu , X. B. Gao , Q. B. Zheng , Carbon 2024, 223, 118976.

[30] L. Chen , X. Mu , Y. Guo , H. Lu , Y. Yang , C. Xiao , Q. Hasi , J. Colloid Interface Sci. 2022, 626, 35. 35780550 10.1016/j.jcis.2022.06.143

[31] Y. Cui , Q. Shi , Z. Liu , J. Lv , C. Wang , X. Xie , S. Zhang , Chem. Eng. J. 2023, 472, 144701.

[32] D. Jiang , J. Zhang , S. Qin , Z. Wang , K. A. S. Usman , D. Hegh , J. Liu , W. Lei , J. M. Razal , ACS Nano 2021, 15, 5000. 33635074 10.1021/acsnano.0c09959

[33] S. Guo , S. Zhang , H. Li , S. Liu , J. J. Koh , M. Zhou , Z. Sun , Y. Liu , H. Qu , Z. Yu , Y. Zhang , L. Yang , W. Chen , C. He , C. Lee , D. Mao , S. K. Ravi , Y. Lai , S. C. Tan , Matter 2025, 8, 101785.

[34] C. H. Kim , H. J. Youn , H. L. Lee , Cellulose 2015, 22, 3715.

[35] S. Zhang , Y. Zhou , A. Libanori , Y. Deng , M. Liu , M. Zhou , H. Qu , X. Zhao , P. Zheng , Y.‐L. Zhu , J. Chen , S. C. Tan , Nat. Electron. 2023, 6, 338.

[36] T. Fujiyabu , X. Li , U.‐I. Chung , T. Sakai , Macromolecules 2019, 52, 1923.

[37] B. G. Amsden , Macromolecules 2022, 55, 8399.

[38] M. Gao , C. K. Peh , L. Zhu , G. Yilmaz , G. W. Ho , Adv. Energy Mater. 2020, 10, 2000925.

[39] Y. Zhang , Q. Zhong , Q. Huang , M. Hu , F. He , Y. Li , Z. Wang , Adv. Funct. Mater. 2024, 34, 2408554.

[40] M. Wu , Y. Wei , Y. Zhu , Y. Bai , Y. Wang , X. Wang , S.‐H. Ho , W. Wang , R. Li , Adv. Funct. Mater. 2024, 34, 2410729.

[41] S. Cheng , E. He , P. Zhang , R. S. Sutar , S. K. Kannan , S. K. Balu , H. Zhao , R. Xing , S. Liu , Adv. Funct. Mater. 2025, 2423011.

[42] Z. Wu , J. Li , S. Zhang , J. Yan , J. Gao , N. Zheng , H. Xue , J. Colloid Interface Sci. 2022, 622, 169. 35490620 10.1016/j.jcis.2022.04.074

[43] Q. Su , Z. Wu , X. Huang , J. Yan , L. Tang , H. Xue , J. Gao , Int. J. Biol. Macromol. 2024, 260, 129403. 38219946 10.1016/j.ijbiomac.2024.129403

