# Supplementary material for: Natural Fiber@MXene‐Engineered Chitosan Aerogels: Thermodynamic‐Transport Synergy for Solar‐Driven Hypersaline Interfacial Evaporation
Source: Adv Sci (Weinh). 2025 May 20;12(30):e05944. doi: 10.1002/advs.202505944 (PMC12376629; doi:10.1002/advs.202505944)
Supplement: Supplementary file 1 — Supporting Information [file ADVS-12-e05944-s001.docx]

**Supporting information**

Qin Su^a^, Haidi Wu^a^, Suyang Hou^a^, Liping Ye^a^, Yifan Feng^a^, Longjuan Lu^a^, Biwang Pan^a^, Wancheng Gu^a^, Longcheng Tang^b^, Xuewu Huang^c^, Huaiguo Xue*^a^,

Jiefeng Gao*^a^

^a^ School of Chemistry and Chemical Engineering, Yangzhou University, No 180, Road Siwangting, Yangzhou, Jiangsu, 225002, China.

^b^ Key Laboratory of Organosilicon Chemistryand Material Technology of Ministry of Education, Hangzhou Normal University，Building 22, Qinyuan, No.2318, Yuhangtang Road, Cangqian Street, Yuhang District, Hangzhou 311121, People’s Republic of China.

^c^ Testing Center, Yangzhou University, Yangzhou, Jiangsu Province 225002, China

*Corresponding authors: chhgxue@yzu.edu.cn; jfgao@yzu.edu.cn.

**Preparation of Ti₃C₂T_X_ (MXene) Dispersion**

To prepare the Ti₃C₂T_X_ dispersion, 1 g of LiF was added to a Teflon container containing 20 mL of 9 M HCl. This mixture was magnetically stirred for 15 minutes to create the etching solution. Following this, 1 g of Ti₃AlC₂ (MAX phase) powder was introduced and stirred at 35 °C for 24 hours. After the reaction, the mixture was washed with deionized water at 3500 rpm until the supernatant pH exceeded 6. The supernatant was then combined with the precipitate and subjected to ultrasound treatment at 285 W for 1 hour using an ultrasonic cell crusher (ultrasonic cell disruptor, JY98-IIIDN, NINGBO SCIENTZ BIOTECHNOLOGY CO. LTD.). Finally, the solution was centrifuged at 3500 rpm for 1 hour to isolate the MXene (Ti₃C₂Tx) solution, yielding MXene nanosheets approximately 200-500 nm in size.

**Contact angle (CA) measurements**

The wettability of samples was determined by an optical angle measuring device (OCA20). The diffusion and adsorption processes of 5 μL deionized water dropped onto aerogels surface were captured by a high-speed camera. The CA value was the mean of the three measurements on different place of the CMKAs surface.

**Photothermal performance measurements**

Different samples were positioned directly under the Xenon lamp at varying solar intensities. The surface temperature of the samples was monitored in real-time using an infrared (IR) thermal imaging device (TiS10, Fluke).

**Salt resistance and different anisole-in-water emulsion evaporation test**

Solutions of sodium chloride (NaCl) with varying mass fractions of 3.5, 10, 15, and 20 wt% were employed to mimic seawater with different salinities. The evaporation apparatus, containing different samples, was utilized in the NaCl solutions prepared for this purpose.

Four organic solvents (n-heptane, toluene, cyclohexane and anisole) insoluble in water were selected to prepare 2 vol% emulsion. The water and organic solvent were combined in a ratio of 50:1, and a precise quantity of the surfactant sodium dodecyl sulfonate (SDS) was incorporated at a concentration of 0.1 mg/ml. The resulting mixture underwent magnetic stirring at a rotational speed of 1000 r/min for 24 hours to achieve a stable emulsion. The method of evaporation test is consistent with the above method, in which simulated seawater is replaced with emulsion.

**Compression performance test**

Compression performance testing of different aerogels was conducted using a universal testing machine, with a loading speed set at 20 mm/min. Stress variations were recorded at different strains.


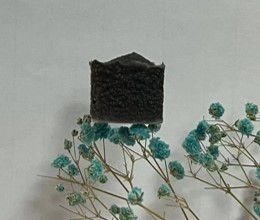


**Fig.S1** Photograph of CMK_0.5_A placed on a bouquet of babysbreath.


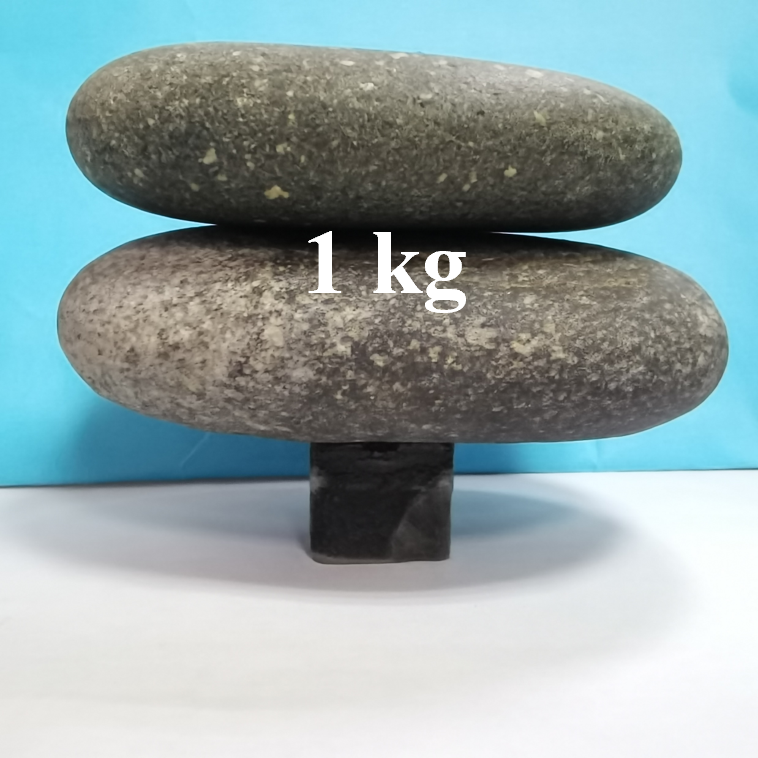


**Fig. S2** Photograph of CMK_0.5_A when subjected to a weight of 1 kg.

**Fig. S3** Compressive stress along the vertical direction during loading and unloading cycles with the step-increased strain of 10% for I- CMKA.


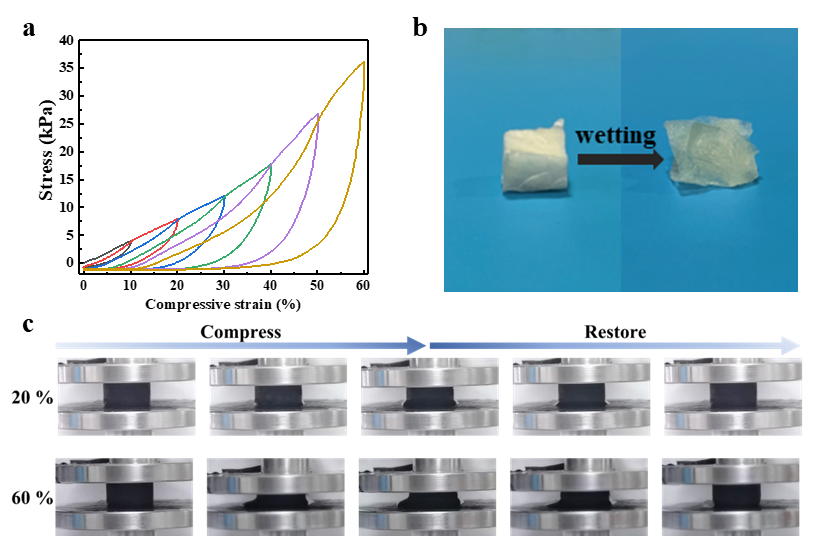


**Fig. S4** (a) Compressive stress of CMK_0.5_A in the wetted state during loading and unloading cycles with the step-increased strain of 10%. (b) The wetting process of pure chitosan aerogel. (c) Photographs of the wetted CMK_0.5_A during compressing and releasing with the strain of 20% and 60%.


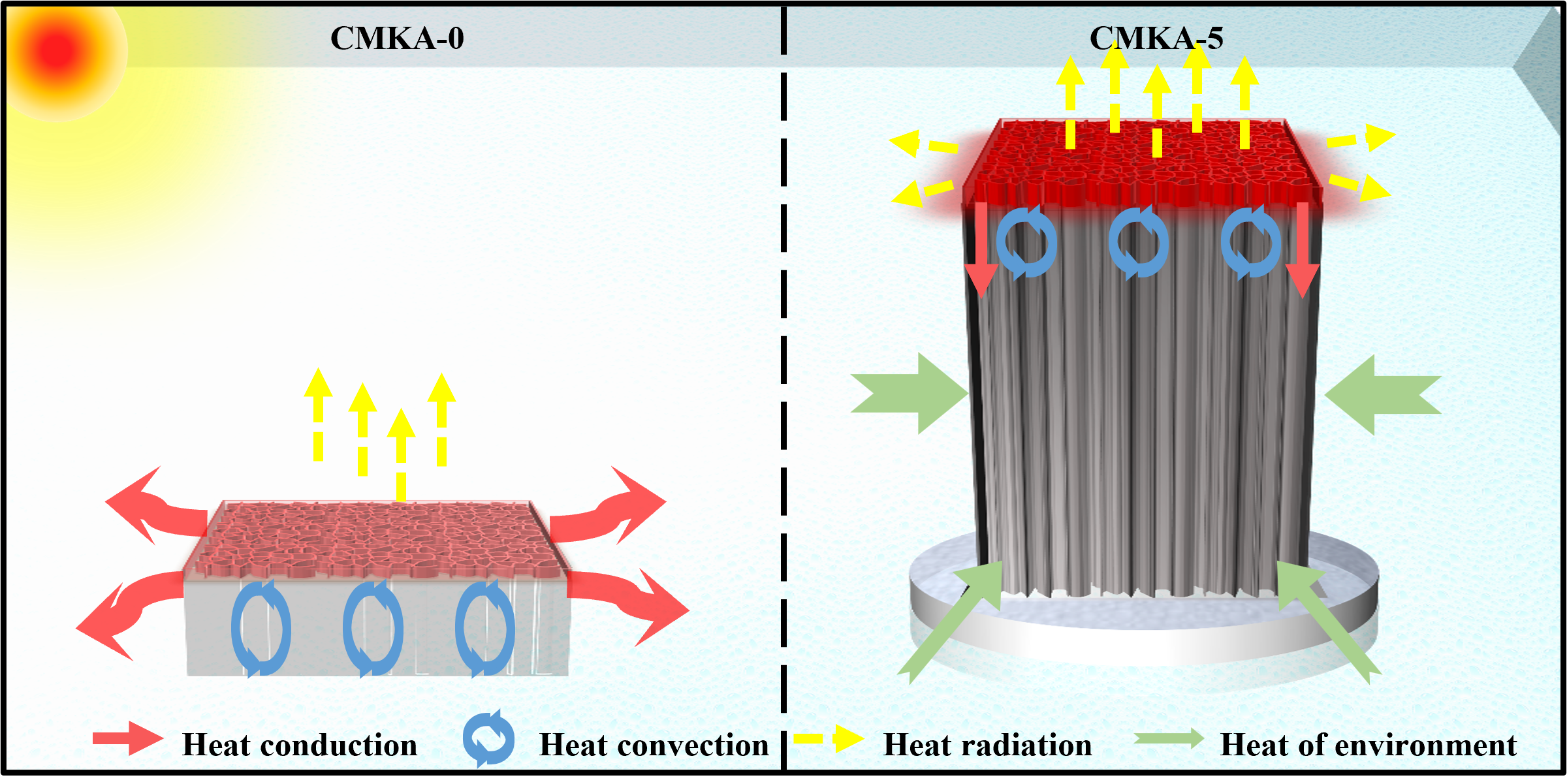


**Fig. S5** Heat loss and utilization of different height CMKA during interfacial evaporation.


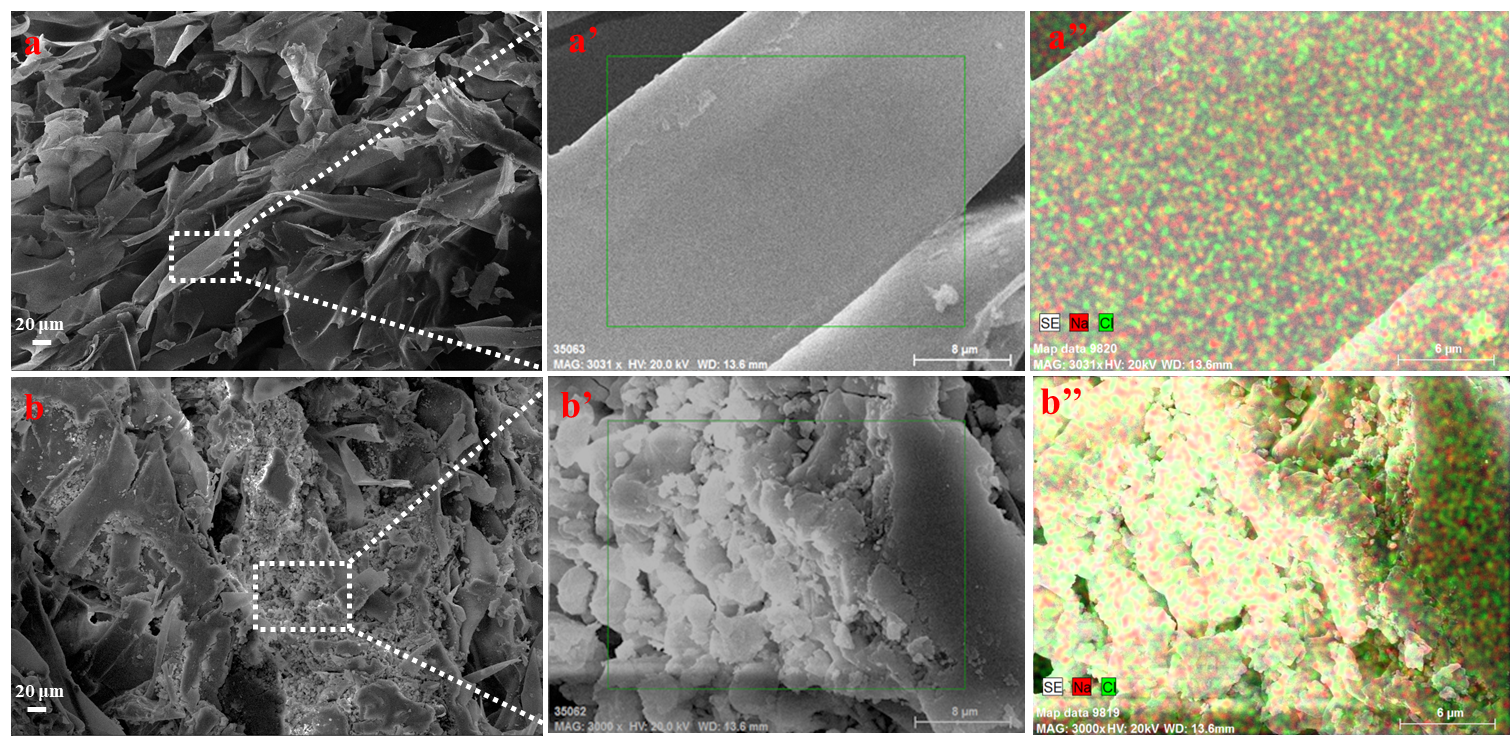


**Fig. S6** (a, a’, a’’) Images, local magnifications and distributions of Na^＋^ and Cl^-^ elements on the surface of CMKA-5 and (b, b’, b’’) I-CMKA-5 after 6 h of irradiation in 20 wt.% brine. (The red dots are Na^＋^, the green dots are Cl^-^)


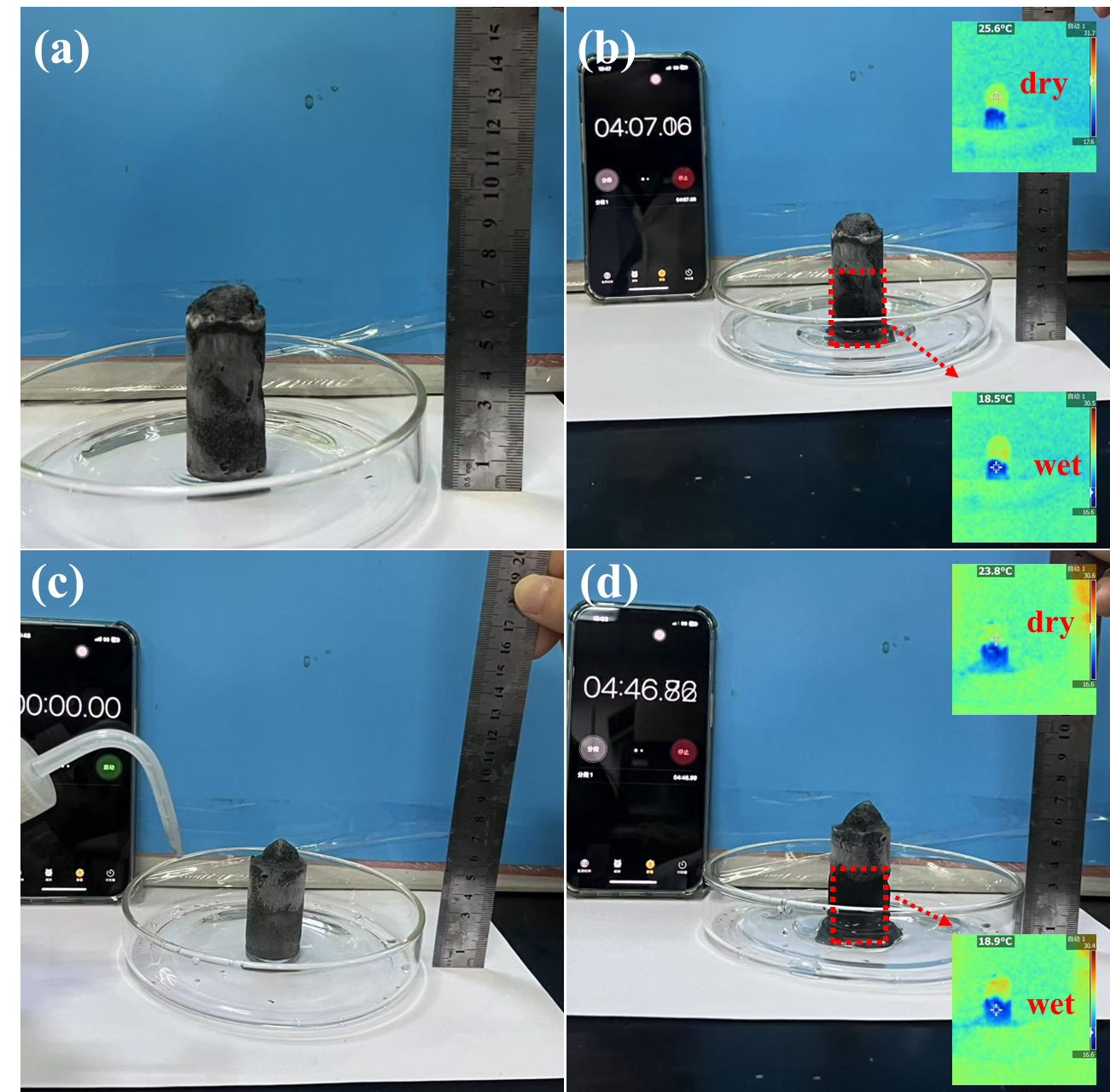


**Fig. S7** Mass transport test for CMKA with aligned pores (a, b) and I-CMKA with random pores (c, d). (The time for water to wet the evaporator from bottom to top to a height of approximately 4cm is tested, and the height is further determined by an infrared imager.)


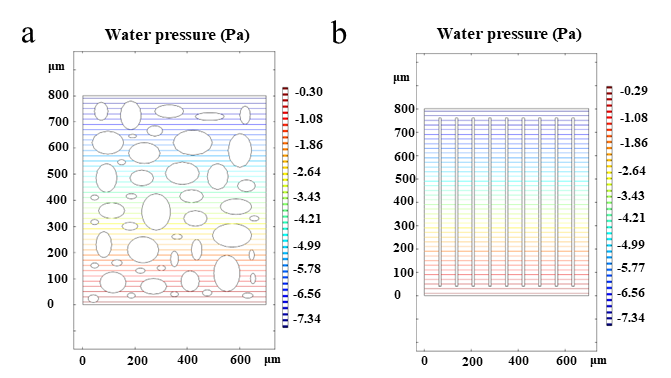


**Fig. S8** Simulated water pressure distributions inside (a) I-CMKA-5 and (b) the CMKA-5 during evaporation.


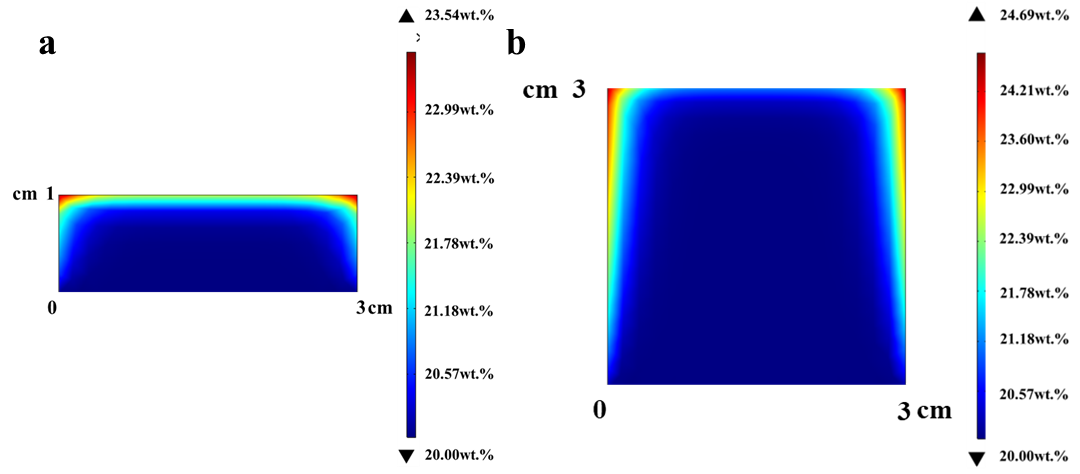


**Fig. S9** Simulated salt concentration distribution of CMKA-1 and CMKA-3 cross section during evaporation. (0 cm at the bottom of the evaporator is in direct contact with the water)


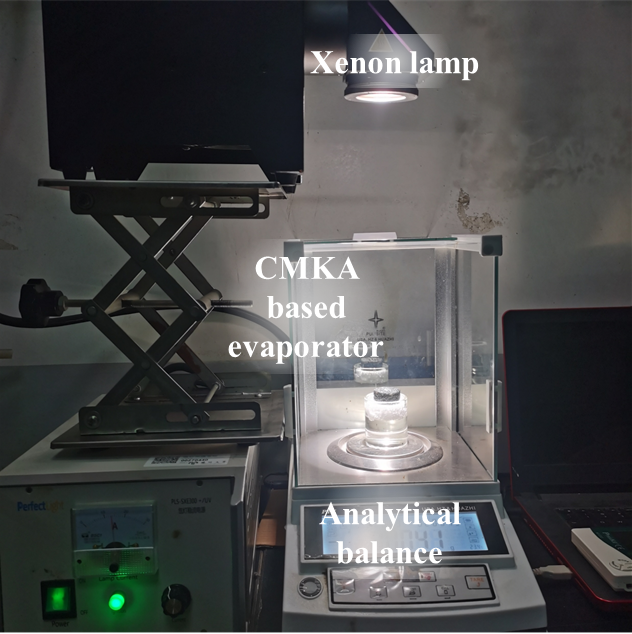


**Fig. S10** Photograph of the laboratory evaporation device.


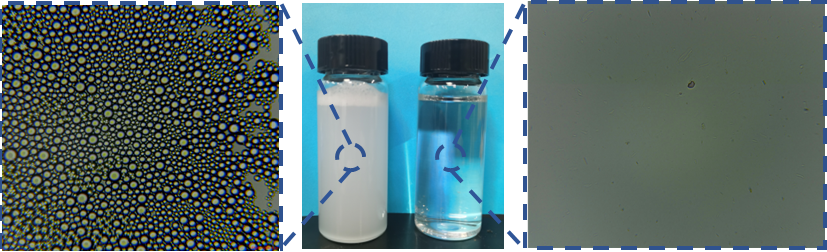


**Fig. S11** Physical and optical microscope images of cyclohexane -in-water emulsion before and after evaporation.


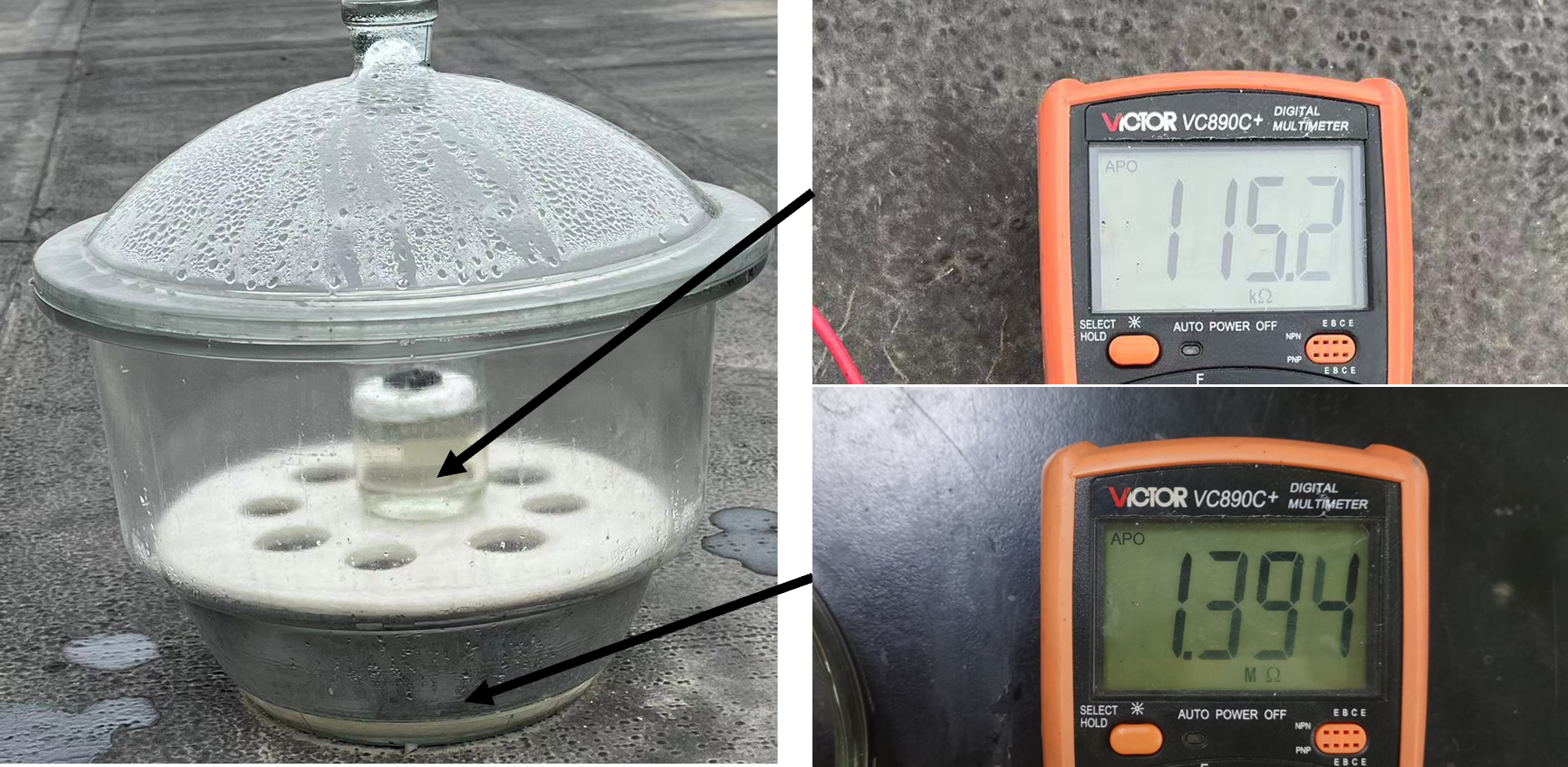


**Fig. S12** Physical drawings of actual outdoor light evaporation units and simulated conductivity of seawater and condensate.

**Heat loss calculation**

**Conductive heat** flux (J_cond_) from CMKA to the bulk water can be determined based on the following equation:

J_cond_ = C m ΔT_bulk_ (2)

Where C is the specific heat capacity of water (4.2 kJ ℃^-1^ kg^-1^), m represents the weight of bulk water, and ΔT_bulk_ represents the increased temperature of the bulk water after stable steam generation.

**Radiative heat** transfer can be calculated using the Stefan-Boltzmann equation:

Φ = ε A σ (T_CMKA_^4^–T_environment_^4^) (3)

Where Φ denotes the heat flux, ε is the emissivity, A is the area of the evaporation surface (1×10^-4^ m^2^), σ is the Stefan-Boltzmann constant (5.67x10^-8^ W m^-2^ K^-4^), T_CMKA_ and T_environment_ are the temperatures of CMKA and environment, respectively. Since CMKA has a relatively high light absorption, it can be treated as a black-body in thermal equilibrium with an ε of 1.

**Convective heat** transfer, J_conv_ can be calculated by the Newton’s law of cooling:

J_conv_ = h A (T_CMKA_ - T_environment_) (4)

Where h is the convective heat transfer coefficient (5-10 W m^-2^ K^-1^).


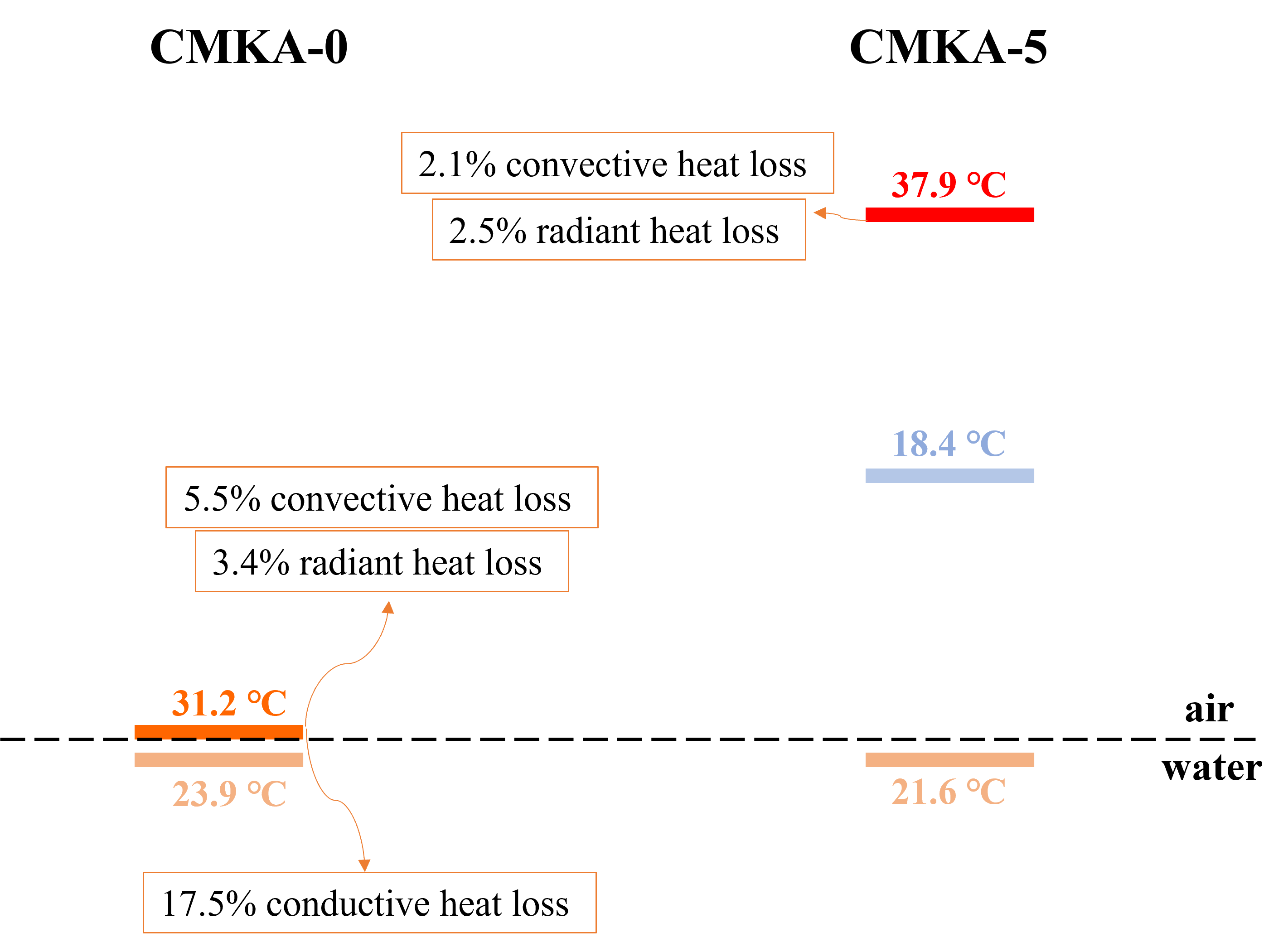


In this calculation, the only energy input is one sunlight irradiation in one hour:

E_in_ = I_in_ ×A×t (5)

The heat loss of CMKA-0 is radiant heat loss (3.4%), conductive heat loss (17.5%) and convective heat loss (5.5%).

We do not calculate the conduction heat loss of CMKA-2 and CMKA-5, considering that the conduction heat at the top of evaporators and the heat in the environment together constitute the evaporation of the side wall.

$\eta=\frac{H_{eva}\cdot m_{eva}}{Q_{abs}}$ (6)

$Q_{abs}=Q_{solar}+Q_{envir}$ (7)

$Q_{solar}=\alpha\cdot S_{Top}\cdot I_{solar}$ (8)

$Q_{envir}=Q_{rad}+Q_{conv}$ (9)

$Q_{rad}=\varepsilon\cdot\sigma\cdot\left( T_{envir}^{4}-T_{side}^{4} \right)\cdot S_{side}+\varepsilon\cdot\sigma\cdot(T_{envir}^{4}-T_{top}^{4})\cdot S_{top}$ (10)

$Q_{conv}=h\cdot\left( T_{envir}-T_{side} \right)\cdot S_{side}+h\cdot(T_{envir}-T_{top})\cdot S_{top}$ (11)

Based on the above various calculations, the following table is obtained, and then the evaporation efficiency of evaporators with different exposure heights is obtained.

| Height cm | T_top_  K | T_side_  K | Q_rad,top_  W | Q_rad,side_  W | Q_conv,top_ W | Q_conv,side_ W |
| --- | --- | --- | --- | --- | --- | --- |
| 0 | 304.2 | / | -0.0034 | / | -0.0055 | / |
| 2 | 307.5 | 293.4 | -0.039 | 0.048 | -0.067 | 0.038 |
| 5 | 310.9 | 291.4 | -0.0054 | 0.167 | -0.092 | 0.300 |

**COMSOL simulation**

The software COMSOL Multiphysics 5.5 was utilized to simulate temperature distribution, isotherm, and salt distribution in CMKAs. A simulation analysis model based on heat distribution and salt concentration distribution of CMKA was established. The simulation involves a dilute material flow, liquid heat transfer module.

**Simulation of heat distribution**

The two-dimensional rectangle was selected as the simulation model of the CMKA cross-section direction. The radius of the three-dimensional simulation analysis model was 1.5cm, and the model height was determined with the height of evaporator above the water surface. A constant solar irradiation of 1 kW m^−2^ is applied to drive the evaporating top surface. The water flux into the evaporator is equal to the outflow flux caused by evaporation, and the heat loss on the top and side of the evaporator is caused by heat convection, heat radiation and heat carried away by evaporation. The simulation is performed by solving the following equations:

$\text{ρ∙}\text{C}_{\text{p,tot}}\text{u×}\text{∇}\text{T+}\text{∇}\text{×q=Q+}\text{Q}_{\text{eva}}\text{+}\text{Q}_{\text{c}}\text{+}\text{Q}_{\text{e}}$ (12)

$\text{Q}_{\text{eva}}\text{=-}\text{h}_{\text{evap}}\text{m}_{\text{eva}}$ (13)

$\text{Q}_{\text{c}}\text{=-}\text{h}_{\text{c}}\text{(T-}\text{T}_{\text{e}}\text{)}$ (14)

$\text{Q}_{\text{e}}\text{=-}\text{E}_{\text{m}}\text{(}\text{T}^{\text{4}}\text{-}\text{T}_{\text{e}}^{\text{4}}\text{)}$ (15)

where 𝜌 is the density, C_p,tot_ is the total heat capacity at constant pressure, q is the conductive heat flux, k_eff_ is the effective thermal conductivity, u is the velocity field, Q_evap_ is the latent heat source in CMKA, h_evap_ is the vaporization enthalpy, m_eva_ is the evaporation flux on the evaporating surface, Q_c_ is the heat convection on the surface, hc is the convection coefficient (10 W m^−2^ K^−1^), Q_e_ is the heat radiation on the surface, Em is the surface emissivity (1.0), 𝜎 is the Stefan–Boltzmann constant (5.67 × 10^−8^ W m^−2^ K^−4^), T_e_ is the environment temperature (298.15 K), Q is the energy input on the top surface of evaporator, q_i_ is the solar intensity (1 kW m^−2^) applied to the evaporator, and 𝛼 is the solar absorptance efficiency (95%) of CMKA.

**Simulation of salt distribution**

To simulate the salt and heat distribution of CMKA-based evaporator, the mass flux of NaCl (J_eva_) and mass loss was applied on the evaporation interface:

$\text{J}_{\text{eva}}\text{=}\frac{\text{m}_{\text{eva}}\text{∙c}}{\text{ρ}}$ (16)

$\text{m}_{\text{eva}}\text{=-}\text{k}_{\text{m}}\frac{\text{M}}{\text{R}}\text{(}\frac{\text{P}_{\text{w}}^{\text{v,sat}}}{\text{T}}\text{-}\text{H}_{\text{R}}\frac{\text{P}_{\text{e}}^{\text{v,sat}}}{\text{T}_{\text{e}}}\text{)}$ (17)

$\text{P}_{\text{x}}^{\text{v,sat}}\text{=}\text{P}_{\text{0}}\text{exp(}\frac{\text{h}_{\text{eva}}}{\text{R}}\text{M(}\frac{\text{1}}{\text{T}_{\text{0}}}\text{-}\frac{\text{1}}{\text{T}}\text{))}$ (18)

Where M represents the molar mass of water, given as 18 × 10^−3^ kg mol^−1^, the universal gas constant is denoted as R, with a value of 8.314 J mol^−1^ K^−1^, The pressures of water vapor at the surface temperature T and the environmental temperature T_e_ are represented as $\text{P}_{\text{w}}^{\text{v,sat}}$ and$\text{P}_{\text{e}}^{\text{v,sat}}$, respectively. Additionally, H_R_ denotes the relative humidity, while km signifies the convective mass transfer coefficient.​P_0_ is the standard pressure, and T_0_ refers to the boiling point of water.

**Table. S1** Comparison of evaporation performance of CMKA-based evaporator with other reported evaporators.

| Materials | Evaporation rate  (kg·m^-2^·h^-1^) | Efficiency (%) | Ref. |
| --- | --- | --- | --- |
| MXene/gelatin aerogel | 1.70 | 90.3 | [1] |
| SM-Ti_3_C_2_T_x_/PVA Aerogels | 1.90 | 88.0 | [2] |
| Janus CNT sheets | 3.00 | 95.0 | [3] |
| MXene/wood aerogel | 1.77 | 92.6 | [4] |
| CNTs/CMC-Na | 2.77 | 90.2 | [5] |
| clay/graphene aerogels | 4.11 | 95.0 | [6] |
| Ag-MnO_2_/graphene aerogel | 6.46 | 90.2 | [7] |
| P(AM-DMDAAC)/GO | 3.65 | 98.9 | [8] |
| partially carbonized Enteromorpha aerogel | 1.87 | 92.0 | [9] |
| cellulose/CNT aerogel | 1.81 | 92.5 | [10] |
| CNT/ PI fibers | 2.08 | 100.0 | [11] |
| CNTs-SA-aerogel | 1.73 | 91.2 | [12] |
| CNFs-COO−/CTS (CC) aerogel | 4.21 | 93.2 | [13] |
| carbon dot-modified starch aerogel | 2.29 | 93.5 | [14] |
| PI-MWCNT/ hollow glass microspheres composite aerogel | 1.51 | 88.8 | [15] |
| KFs-MXene | 1.47 | 90.4 | [16] |
| octadecane/ppy aerogel | 2.62 | 92.7 | [17] |
| MXene aerogel | 3.30 | 86.0 | [18] |
| carbon aerogel | 1.78 | 85.0 | [19] |
| PAN/PVDF aerogel | 1.46 | 93.2 | [20] |
| Chitosan /MXene/ Kapok fiber composite aerogel | 2.74 | 94.5 | This work |

**References**

[1] N Xue, H Cui, W Dong, et al. Multifunctional hydrophilic MXene/Gelatin composite aerogel with vertically aligned channels for efficient sustainable solar water evaporation and Oil/Water separation [J]. Chemical Engineering Journal, 2023, 455: 140614.

[2] H Zhang, X Shen, E Kim, et al. Integrated Water and Thermal Managements in Bioinspired Hierarchical MXene Aerogels for Highly Efficient Solar-Powered Water Evaporation [J]. Advanced Functional Materials, 2022, 32(19): 2111794.

[3] C Wang, S Zhou, C Wu, et al. Janus carbon nanotube sponges for highly efficient solar-driven vapor generation [J]. Chemical Engineering Journal, 2023, 454: 140501.

[4] H Gao, N Bing, Z Bao, et al. Sandwich-structured MXene/wood aerogel with waste heat utilization for continuous desalination [J]. Chemical Engineering Journal, 2023, 454: 140362.

[5] Z Jin, M Zhang, H Mei, et al. 3D-printed chiral torsion Janus evaporator with enhanced light utilization towards ultrafast and stable solar-water desalination [J]. Carbon, 2023, 202: 159-68.

[6] M Ding, H Lu, Y Sun, et al. Superelastic 3D Assembled Clay/Graphene Aerogels for Continuous Solar Desalination and Oil/Organic Solvent Absorption [J]. Advanced Science, 2022, 9(36): 2205202.

[7] Y Tian, C Du, S Yong, et al. Catalysis-involved 3D N-doped graphene aerogel achieves a superior solar water purification rate and efficiency [J]. Chemical Engineering Journal, 2023, 453: 139793.

[8] X Cheng, Y Kong, Y Gao, et al. One-step construction of P(AM-DMDAAC)/GO aerogel evaporator with Janus wettability for stable solar-driven desalination [J]. Separation and Purification Technology, 2022, 303: 122285.

[9] C Wang, Y Wang, M Yan, et al. Highly efficient self-floating jellyfish-like solar steam generators based on the partially carbonized Enteromorpha aerogel [J]. Journal of Colloid and Interface Science, 2023, 630: 297-305.

[10] M K Alam, M He, W Chen, et al. Stable and Salt-Resistant Janus Evaporator Based on Cellulose Composite Aerogels from Waste Cotton Fabric [J]. ACS Applied Materials & Interfaces, 2022, 14(36): 41114-21.

[11] Y Ren, R Lian, Z Liu, et al. CNT/polyimide fiber-based 3D photothermal aerogel for high-efficiency and long-lasting seawater desalination [J]. Desalination, 2022, 535: 115836.

[12] N An, Y Jiang, Z Wang, et al. Efficient water purification and desalination using hydrogel and aerogel solar evaporators based on different carbon materials [J]. Separation and Purification Technology, 2022, 301: 122003.

[13] J He, N Li, S Wang, et al. Efficient Solar-Powered Interfacial Evaporation, Water Remediation, and Waste Conversion Based on a Tumbler-Inspired, All-Cellulose, and Monolithic Design [J]. Advanced Sustainable Systems, 2022, 6(10): 2200256.

[14] X Xu, Q Chang, C Xue, et al. A carbonized carbon dot-modified starch aerogel for efficient solar-powered water evaporation [J]. Journal of Materials Chemistry A, 2022, 10(21): 11712-20.

[15] S Wang, Y Niu, W Mu, et al. Robust hollow glass microspheres-based solar evaporator with enhanced thermal insulation performance for efficient solar-driven interfacial evaporation [J]. Materials Today Chemistry, 2022, 26: 101042.

[16] L Chen, X Mu, Y Guo, et al. MXene-doped kapok fiber aerogels with oleophobicity for efficient interfacial solar steam generation [J]. Journal of Colloid and Interface Science, 2022, 626: 35-46.

[17] L Geng, L Li, H Zhang, et al. Interfacial solar evaporator synergistic phase change energy storage for all-day steam generation [J]. Journal of Materials Chemistry A, 2022, 10(29): 15485-96.

[18] X Che, W Zhang, L Long, et al. Mildly Peeling Off and Encapsulating Large MXene Nanosheets with Rigid Biologic Fibrils for Synchronization of Solar Evaporation and Energy Harvest [J]. ACS Nano, 2022, 16(6): 8881-90.

[19] H Zhang, L Li, N He, et al. Bioinspired hierarchical evaporator via cell wall engineering for highly efficient and sustainable solar desalination [J]. EcoMat, 2022, 4(5): e12216.

[20] W Yao, X Li, X Zhu, et al. Thermal-localized and salt-resistant polyacrylonitrile/polyvinylidene fluoride aerogel for efficient solar desalination [J]. Desalination, 2022, 532: 115751.
